# Eu(III) and Cm(III) Complexation by the Aminocarboxylates NTA, EDTA, and EGTA Studied with NMR, TRLFS, and ITC—An Improved Approach to More Robust Thermodynamics

**DOI:** 10.3390/molecules28124881

**Published:** 2023-06-20

**Authors:** Sebastian Friedrich, Claudia Sieber, Björn Drobot, Satoru Tsushima, Astrid Barkleit, Katja Schmeide, Thorsten Stumpf, Jerome Kretzschmar

**Affiliations:** 1 Helmholtz-Zentrum Dresden-Rossendorf, Institute of Resource Ecology, 01328 Dresden, Germany; 2 International Research Frontiers Initiative, Institute of Innovative Research, Tokyo Institute of Technology, Meguro, Tokyo 152-8550, Japan

**Keywords:** spectroscopy, calorimetry, molecular structure, high-affinity ligand, complex formation constant

## Abstract

The complex formation of Eu(III) and Cm(III) was studied via tetradentate, hexadentate, and octadentate coordinating ligands of the aminopolycarboxylate family, viz., nitrilotriacetate (NTA^3−^), ethylenediaminetetraacetate (EDTA^4−^), and ethylene glycol-bis(2-aminoethyl ether)-*N*,*N*,*N*′,*N*′-tetraacetate (EGTA^4−^), respectively. Based on the complexones’ p*K*_a_ values obtained from ^1^H nuclear magnetic resonance (NMR) spectroscopic pH titration, complex formation constants were determined by means of the parallel-factor-analysis-assisted evaluation of Eu(III) and Cm(III) time-resolved laser-induced fluorescence spectroscopy (TRLFS). This was complemented by isothermal titration calorimetry (ITC), providing the enthalpy and entropy of the complex formation. This allowed us to obtain genuine species along with their molecular structures and corresponding reliable thermodynamic data. The three investigated complexones formed 1:1 complexes with both Eu(III) and Cm(III). Besides the established Eu(III)–NTA 1:1 and 1:2 complexes, we observed, for the first time, the existence of a Eu(III)–NTA 2:2 complex of millimolar metal and ligand concentrations. Demonstrated for thermodynamic studies on Eu(III) and Cm(III) interaction with complexones, the utilized approach is commonly applicable to many other metal–ligand systems, even to high-affinity ligands.

## 1. Introduction

Nitrilotriacetic acid (H_3_NTA), ethylenediaminetetraacetic acid (H_4_EDTA), and ethylene glycol-bis(2-aminoethyl ether)-*N*,*N*,*N*′,*N*′-tetraacetic acid (H_4_EGTA) are typical representatives of aminopolycarboxylic acids. They easily form very stable complexes with various metal ions; hence, they are commonly referred to as complexones. Their coordination compounds with a wide range of metals from all over the periodic table have been and are still of interest for several reasons, e.g., as reference and model compounds or in specific applications (see below) for complex distinct metal ions.

In this paper, the subject of interest is the complexones’ chemical behavior towards trivalent ions of two members of the lanthanide and actinide series, using an advanced multi-method approach which is applicable to many other metal–ligand systems. Trivalent europium, Eu(III), is commonly used as a non-radioactive chemical analog to study trivalent actinides, An(III), owing to its excellent luminescence properties which make it a good probe for time-resolved laser-induced fluorescence spectroscopy, TRLFS. Trivalent curium, Cm(III), also shows superb luminescence and constitutes a direct representative of An(III), and is present in significant amounts in spent nuclear fuel.

EDTA and NTA are especially regularly utilized in the nuclear industry as decontamination agents that are used, for instance, during the operation or decommissioning of nuclear power plants [1,2]. Thus, high volumes of decontamination solutions are generated as secondary waste that is finally co-disposed with radioactive wastes in repositories for low- and intermediate-level radioactive waste (L/ILW) [3,4,5,6,7]. In the worst case of water ingress, these organic complexing agents have the potential to enhance radionuclide migration from radioactive wastes.

In mining and industrial regions, increased concentrations of lanthanides in both the environment and population are reported in the literature. For example, in tributaries of the Chinese Yellow River, the total amount of cerium, Ce, was determined to be 3380 µg L^−1^, and the overall amount of lanthanides was determined to be 8507 µg L^−1^ [8]. The concentrations of Ce and the sum of all lanthanides in the blood of inhabitants of the mining area in the southwest Fujian Province were determined to be 603 and 645 µg L^−1^, respectively [9]. An overview was previously conducted by Heller et al. [10]. (Poly)aminopolycarboxylic acids are suitable decorporation agents for such heavy metals. From the complexones analyzed in this paper, NTA and EDTA have already been tested as decorporation agents [11,12,13,14]. Another example expanding this group is diethylenetriaminepentaacetic acid (H_5_DTPA), which is currently the decorporation agent of choice against plutonium, americium, and curium [14,15]. To develop more effective decorporation strategies in the future, a fundamental understanding of the complex formation reaction between the metal ion and the ligand is crucial.

With regard to the used approach demonstrated in this paper, well-characterized complexone–metal complexes are frequently used in analytical experiments (of related disciplines) to determine complex formation constants of high-affinity ligands [16,17] or at least qualitatively show the supremacy of a new developed chelator’s binding capacity [18,19]. The affinity of those ligands is often so high that for a direct measurement of complex formation constants, the concentration regime is below detection limits of most spectroscopic techniques. The thermodynamic characterization is therefore carried out indirectly via metal ion competition experiments with complexones. Evidently, the robustness of the complexation constants determined in this way depends very much on the accuracy of the characterization of the competing (reference) metal–complexone complexes.

The complexones’ complexation behavior is quite well understood; however, most complex formation constants are based on data that are multiple decades old [20,21,22,23]. Many complex formation constants have been determined using potentiometric measurements [22]; some data have been determined by means of TRLFS [24,25]. Methods for determining complex-formation data have since become more reliable and accurate. Furthermore, the complexation constants for the trivalent f-elements were often also determined indirectly by means of competition experiments involving divalent metal ions, with the limitations already mentioned above.

This study aims not only to provide information on Eu(III) and Cm(III) complexation by NTA, EDTA, and EGTA, but also propose a procedure to achieve results with high accuracy.

Acid dissociation constants for all three complexones are available; however, they are inconsistent, divergent, and sometimes only cover those of the carboxylic groups, disregarding that the amine nitrogen is protonated at least up to near-neutral conditions. For complexation studies at pH values below the ammonium’s corresponding p*K*_a_, its contribution is critical as it has the highest contribution to the brutto complex formation constant (log *β*). Therefore, our studies also comprise the determination of the ligands’ p*K*_a_ values. With this approach, it is possible to directly access interactions between metal ions and high-affinity complexones. An exact determination of p*K*_a_ values allows for a precise speciation model of the corresponding ligands. According to this speciation, the experimental conditions for the metal-to-ligand titration are set up so that the affinity of the ligand is low enough for a direct measurement; that is, a sufficiently low pH providing a well-defined (and easy-to-determine) concentration of H^+^ to compete with the metal ion for the binding sites. Overall, this workflow helps overcome indirect methods—like titration against mercury electrode (pHg method)—increases the reliability of the resulting thermodynamic data, and offers direct accessibility even for high-affinity systems.

## 2. Results and Discussion

The structure of the manuscript follows the requirements for a comprehensive thermodynamic characterization of ligand systems. A prerequisite for obtaining accurate thermodynamic constants for complexone–metal systems is the determination of the ligands’ p*K*_a_ values. NMR was used for this purpose in addition to structure elucidation from the ligands’ perspective. Subsequently, TRLFS was used to check the pH-dependent behavior of the complexone–metal system. Based on the previous results, a suitable pH for concentration titrations was set, at which the ligands’ affinity was reduced, allowing the direct measurement of the formation constants of the complexes. These were then determined using ITC and TRLFS in a complementary manner.

### 2.1. Nuclear Magnetic Resonance Spectroscopy (NMR)

#### 2.1.1. p*K*_a_ Determination from NMR pH Titration Series

**Figure 1:**
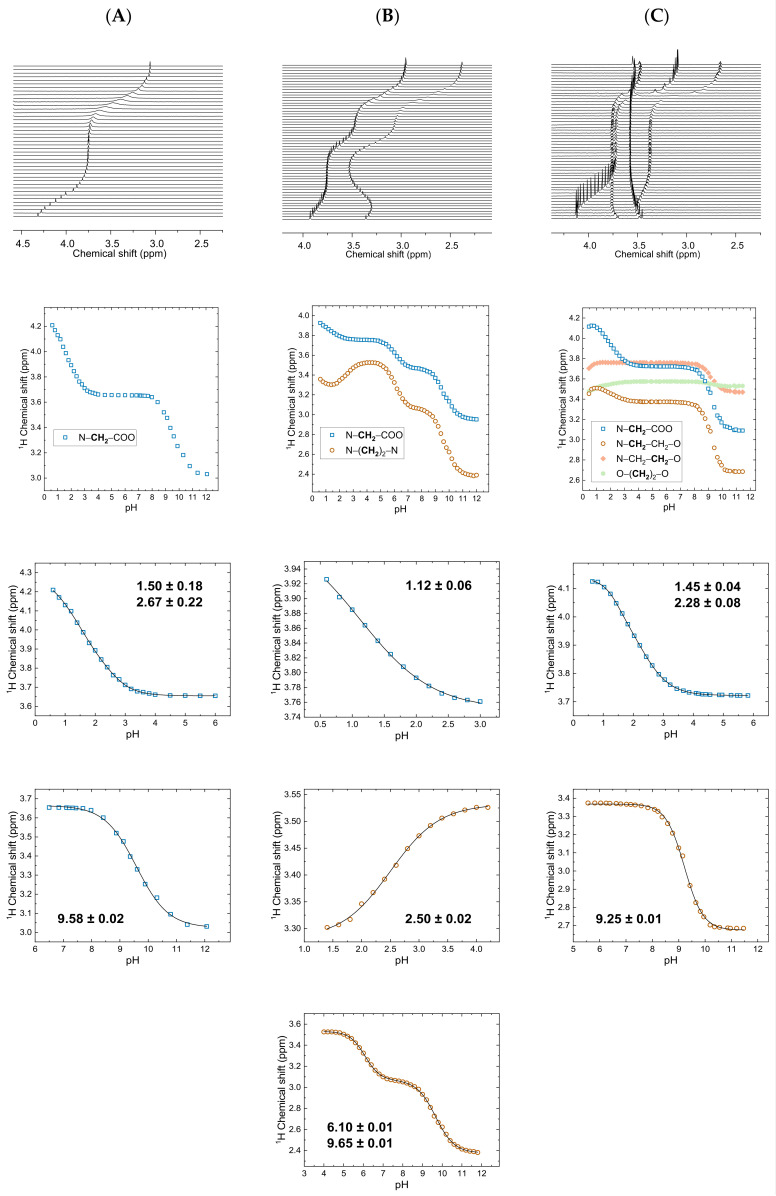
^1^H NMR pH titration spectra (top row) of NTA (**A**), EDTA (**B**), and EGTA (**C**) in the pH range 0.6–12.0, and pH values increasing from bottom to top with increments of 0.1 to 0.4 pH units, along with pH-dependent ^1^H chemical shift data (second row) as well as corresponding sigmoidal (bi-)dose–response fits (black lines) allowing for determining the p*K*_a_ values, respectively, stated with the plots.

As a critical prerequisite for subsequent calculations of reliable thermodynamic quantities of the metal ion complexes, the ligands’ p*K*_a_ values were determined by means of NMR spectroscopy. This allows for the assignment of the protolytic site that the respective H^+^ is abstracted from, by means of the method’s superb structure sensitivity. Figure 1 shows a compilation of how p*K*_a_ values of NTA (Figure 1A), EDTA (Figure 1B), and EGTA (Figure 1C) were determined: based upon the ^1^H NMR pH titration spectra (top row) and their graphical evaluation as *δ*_H_ vs. pH plots (second row), p*K*_a_ values were obtained from the inflection points of sigmoidal (bi-)dose–response fit functions applied to the data points (black lines in the, respectively, partitioned *δ*_H_ vs. pH plots). Table 1 summarizes our obtained figures and compares the values to the available literature data. Spectra and evaluation for a 40 mM NTA series (1 M NaCl) are provided as Supplementary Materials, Figures S1 and S2.

Under very acidic conditions (pH ≤ 2), all three complexones slowly crystallized in their zwitterionic forms (one deprotonated carboxylate per protonated amino group) as revealed by single-crystal X-ray diffraction. It is not surprising that the net-neutral zwitterionic forms are the species of lowest solubility. This, in turn, allows for the conclusion that further protonation yielding the species H_4_NTA^+^, H_5_EDTA^+^, and H_6_EDTA^2+^, as well as H_5_EGTA^+^ and H_6_EGTA^2+^, occurs, if at all, only under extreme acidic conditions (p*K*_a_ ≤ 1). Hence, in Table 1, p*K*_a1_ and p*K*_a2_ refer to proton abstraction from the remaining two protonated carboxyl groups, whereas p*K*_a3_ and p*K*_a4_ refer to the deprotonation of the amino group (ammonium form), respectively. Generic structures for all relevant protolytic ligand species are presented in the Supplementary Materials, Figure S3. Remarkably, compared to NTA and the seemingly more related EGTA, EDTA behaves somewhat differently. As seen from EDTA’s N–*CH*_2_*–CH*_2_–N and EGTA’s N–*CH*_2_–CH_2_–O pH-dependent ^1^H chemical shift values of the methylene groups adjacent to the nitrogen atoms, above pH 4, EDTA clearly shows two inflections, whereas EGTA shows only one inflection (cf. *δ*_H_ vs. pH plots in Figure 1, second row). Apparently, in EGTA, the two ammonium groups are separated far enough to behave independently and hence deprotonate practically simultaneously, whereas in the smaller molecule EDTA, these groups are much closer and hence much more strongly affected by Coulomb repulsion. Consequently, one ammonium group in EDTA already deprotonates at a near-neutral solution, 3.5 logarithmic units below the other site in the same molecule or compared to those of NTA and EGTA.

#### 2.1.2. Eu(III) Complexation by NTA and EGTA

Since EDTA and its metal ion complexes, including those of some lanthanides, have already been extensively studied by NMR spectroscopy [29,30,31], we waived the repetition of such experiments. The molecular representations of all considered complexes of NTA, EDTA, and EGTA are depicted in Figures S8–S11, Supplementary Materials.

Solution ^1^H and ^13^C NMR studies cover two pD-dependent series, comprising samples either equimolar in Eu(III) and NTA, or with a twofold excess in NTA. In the case of the equimolar series, samples above pD 7.0 showed the formation of a white solid. Therefore, these solutions were centrifuged and the obtained clear supernatants were subjected to NMR spectroscopy. In solutions with excess NTA, no precipitation occurred.

**Figure 2:**
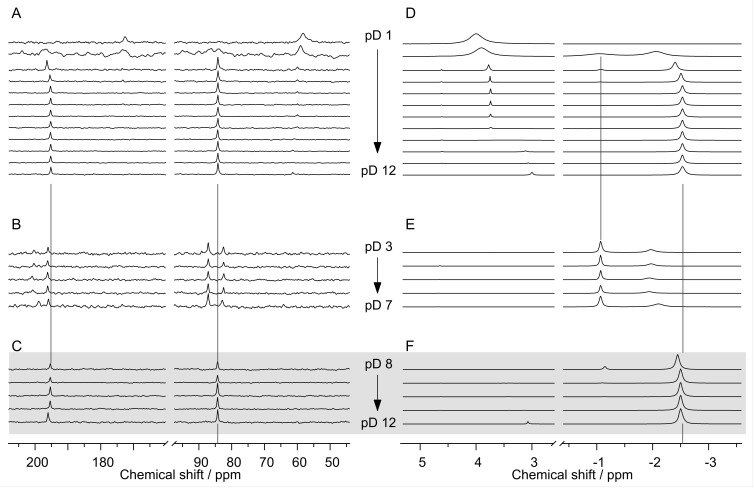
^13^C{^1^H} (**A**–**C**) and ^1^H NMR spectra (**D**–**F**) of D_2_O solutions containing 1 M NaCl, in dependence on pD and NTA/Eu ratio. (**A**,**D**): 15 mM Eu(III), 30 mM NTA, pD 1.0–12.0 (from top); (**B**,**E**): 18 mM Eu(III), 18 mM NTA, pD 3.0–7.0 (from top); (**C**,**F**): supernatants of solutions initially 18 mM Eu(III), 18 mM NTA, pD 8.0–12.0 (from top). pD values are incremented by one integer unit, respectively. The HDO signal was suppressed by a pre-saturation sequence. For clarity, only spectral regions of interest are shown.

The ^1^H and ^13^C NMR spectra acquired for p*K*_a_ determination (Figure 1) now served as blank references. Upon comparison of the corresponding spectra of the blank and the Eu(III)-containing samples of NTA excess series (15 mM Eu(III), 30 mM NTA), at pD 1.0, the peak positions were almost identical. Line widths, however, for ^1^H and ^13^C were less than 3 Hz in the blank but 150 Hz for the ^1^H and carboxyl ^13^C signals and about 280 Hz for the methylene ^13^C signals, respectively (Figure 2A,D, top spectra). These remarkable line broadenings are a clear indication that, for the given concentrations, Eu(III) and NTA interact even in a strongly acidic solution. The line broadening is attributed to two effects; enhanced relaxation due to paramagnetic Eu(III) (f^6^ electron configuration), as can be seen for all signals of the Eu(III)–NTA complexes, and, mainly, the dynamics caused by H^+^ and Eu(III) association and dissociation reactions upon competition for NTA’s functional groups. As of pD 2.0, the interaction between Eu(III) and NTA was further pronounced. As the aforementioned dynamics slowed down, new signals at *δ*_C_ ~ 197 and ~85 ppm as well as at *δ*_H_ ~ −1.1 and ~−2.1 ppm became visible. The pD 3.0 spectrum was characterized by much sharper lines, indicating much slower ligand exchange reactions. As of pD 4.0, the signal at *δ*_H_ ~ −1.1 ppm disappeared, and the remaining signals associated with a Eu(III)–NTA complex remained unaltered up to pD 12.0. Since almost all of the NTA was Eu(III)-bound, we concluded that the spectra of the NTA excess series were dominated by the Eu(III)–NTA 1:2 complex, with corresponding *δ*_C_ 195.3 and 84.3 ppm as well as *δ*_H_ −2.5 ppm.

Solutions prepared with equimolar amounts of Eu(III) and NTA (18 mM each) showed a precipitate formation as of pD 8.0. Corresponding spectra of this series are shown in Figure 2B–F. For samples at pD 3.0–7.0 (Figure 2B,E), the spectra show the same two sets of signals. The absence of signals of unbound NTA indicates that both complexes associated with these two sets of signals possess a Eu(III)/NTA ratio equal to (or greater than) unity. The set comprising the signals at *δ*_C_ 196.2 and 87.2 ppm as well as a rather sharp ^1^H signal at *δ*_H_ −1.1 ppm refers to the Eu(III)–NTA 1:1 complex, and the signals at *δ*_C_ ~ 200.7 and 82.5 ppm as well as the broad ^1^H signal at *δ*_H_ −1.9 ppm are due to the 2:2 complex.

In contrast, the spectra corresponding to the supernatants of the pD 8.0–12.0 samples (Figure 2C,F) resembled those of the 1:2 complex (cf. Figure 2A vs. Figure 2C and Figure 2D vs. Figure 2F). From that, we concluded that for pD > 7.0, upon precipitation of the Eu(OH)_3_ solid, (i) the complex species existing up to pD 7.0 break down by Eu(III) hydrolysis, and (ii) the resulting NTA excess again favors the formation of the 1:2 complex and, hence, prevents further hydrolysis and precipitation, even for millimolar concentrations up to pD 12.0.

In general, all spectra were characterized by Eu(III)’s strong paramagnetic-induced shifts. In all cases, the carboxyl and methylene ^13^C signals were shifted downfield by about 14.1 and 25.7 ppm (1:1), 13.2 and 22.8 ppm (1:2), and 18.6 and 21.0 ppm (2:2), whereas the ^1^H signals were shifted upfield by −4.2 ppm (1:1), −5.6 ppm (1:2), and −5.0 ppm (2:2).

Taking into account the respective line widths of the ^1^H signals, the sharp signals indicated a high symmetry and low molecular dynamics. This was the case for the 1:1 and 1:2 complexes with NTA-chelating Eu(III) in a tetradentate fashion with its three carboxylic groups and the amino nitrogen. On closer inspection, for the other set involving the broad ^1^H signal (*δ*_H_ −1.9 ppm), the signal of the carboxyl carbons was asymmetric and, in fact, comprised two signals with ratio of about 1:2 (see insert Figure S4, Supplementary Materials, along with the single-component ^13^C and ^1^H NMR spectra of the observed three Eu(III)–NTA complexes). This indicates two different types of Eu(III)-bound carboxylic groups in the complex. We therefore propose the structure of the 2:2 complex to contain two Eu(III) ions and two NTA^3−^ ligands, where each Eu(III) is coordinated by one NTA’s three carboxylic groups, and one which also bridges to the other Eu(III). Accordingly, four of the six carboxylate groups are coordinated in a monodentate fashion, and two of the six bridge the Eu(III) ions, hence the two distinct ^13^C carboxylate signals. The broadened methylene ^1^H signal indicates dynamics upon the intramolecular site exchange between the two modes of carboxylate binding, i.e., a monodentate carboxyl becomes bridging, and a bridging carboxylate changes to monodentate coordination.

In 1960, Anderegg described [32] that NTA forms two complexes with Ln(III), i.e., a 1:1 and a 1:2 complex, both acting as tetradentate ligands. However, the existence of higher complexes, such as a 2:3 complex, was excluded, as tetradentate nitrilotriacetate ion forming a sufficiently stable linkage between two metal ions was considered unlikely. By means of NMR, complemented by luminescence spectroscopy and DFT calculation, we were able to unambiguously assign a 2:2 complex. DFT calculation confirmed the NMR-derived structure of the Eu(III)–NTA 2:2 complex (Figure S7, Supplementary Materials). The calculated structure revealed an eight-coordinate environment of either Eu(III), with explicit water molecules strongly associated in a second coordination shell, most likely owing to the strong hydrogen-bonding network involved between the complex’s polar functional groups and surrounding water molecules. In addition, luminescence spectroscopy of the solutions used for NMR (15 and 18 mM Eu(III)) reveal a further set of unique spectroscopic parameters (spectrum and luminescence lifetime; Figure S12, Supplementary Materials) distinct from the Eu(III)–NTA 1:1 and 1:2 complexes. A concentration-dependent series revealed significant abundance (>5%) of the 2:2 complex as of 10^−3^ M Eu(III). For the thermodynamic investigations at 10^−5^ M Eu(III), presented in Section 2.2, this species did not form.

Results obtained for Eu(III) complexation by EGTA were straightforward; corresponding pD-dependent as well as C–H correlation spectra along with signal assignments are provided as Figures S5 and S6, Supplementary Materials. Briefly, at pD 1.0, no interaction between EGTA and Eu(III) was observed as the ^13^C and ^1^H spectra resembled those of EGTA blank (not shown), and signals of the Eu(III)–EGTA complex (cf. pD 3.0–9.0 spectra) were absent. The exclusively existing Eu(III)–EGTA complex persisted for pD 3.0 through (at least) pD 9.0, as evidenced by the virtually identical spectra.

### 2.2. Time-Resolved Laser-Induced Fluorescence Spectroscopy (TRLFS)

The major advantage of f-element luminescence spectroscopy is its sensitivity. Compared to other techniques, metal concentrations below 10^−5^ M can be investigated, which allows researchers to perform speciation studies. In this work, TRLFS was mainly used to determine the distribution of different metal complexes for a thermodynamic analysis. In addition, structural information was obtained to a certain extent.

The luminescence lifetimes of the investigated metal ions are determined via the radiationless de-excitation through various oscillators from the medium or the ligand itself. In aqueous solution, the predominant path for this is through the O-H oscillators from bound water. As a first approximation, a directly proportional dependence of the lifetime on the number of O-H oscillators in the first coordination sphere can be assumed [33]. For Eu(III), this has been previously determined to roughly follow the equation given in Horrocks et al. [33], Equation (1). For Cm(III) a similar empirical relationship, Equation (2), was found by Kimura et al. [34]. The high uncertainty of these formulas can be explained by other oscillators (N-H, C-H, etc.) providing additional de-excitation paths.

(1) $$n\left(H_{2}O\right)\pm 0.5=1.07/\tau [\mathrm{ms}]-0.62$$

(2) $$n\left(H_{2}O\right)\pm 0.5=0.65/\tau [\mathrm{ms}]-0.88$$

In addition, the occurrence and splitting of the ^5^D_0_ → ^7^F_J_ can be used to obtain information about the complex symmetry.

For complex systems, like pH titration series or concentration titrations, maximum information gain can only be achieved using state-of-the-art mathematical tools. Therefore, all TRLFS data were analyzed with PARAFAC as previously described [35].

#### 2.2.1. Eu(III) and Cm(III) Complexation by NTA

**Figure 3:**
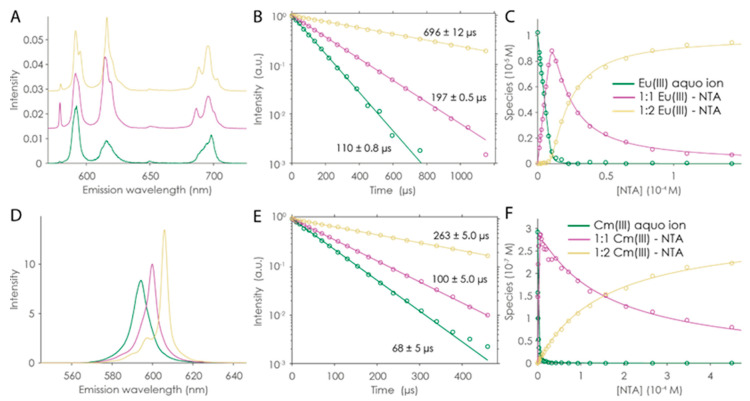
PARAFAC results of TRLFS series of NTA complexation with Eu(III) (**A**–**C**) and Cm(III) (**D**–**F**). Emission spectra (**A**,**D**), luminescence decays (**B**,**E**), and quantum-yield-corrected PARAFAC distributions (symbols) and corresponding speciation (lines) (**C**,**F**).

TRLFS series with varying pH showed similar behavior to the NMR pH series. The initial increase in the intensity of the F_0_ band, followed by its decrease, was an indication that three different Eu(III) species were present in the system. The separation of single species and their relative amounts was conducted using PARAFAC and the corresponding spectra; luminescence decays and distribution are shown in Figure S13, Supplementary Materials. Then, a ligand titration series was conducted to study the complexation of Eu(III) by NTA in more detail at a fixed pH of 6.0 (Figure S14, Supplementary Materials). In Figure 3A–C, the PARAFAC analysis of this TRLFS series (10 µM Eu, *I* = 0.1 M NaCl) is shown. The intense F_0_ transition of the 1:1 complex reflects a reduced symmetry of the Eu(III) center due to the coordination by NTA from only one side. The additional ligand in the 1:2 complex restored the symmetry partially and the F_0_ band intensity decreased. The increasing luminescence lifetime with complexation agreed well with a decreasing number of water molecules in the Eu(III) coordination sphere according to Equation (1). For the 1:1 and 1:2 complexes, 4.8 ± 0.5 and 0.9 ± 0.5 remaining water molecules were determined, respectively. The obtained luminescence lifetimes for the 1:1 and 1:2 species, (197 ± 0.5) and (696 ± 12) µs, are in agreement with reported 190 and 725 µs [36].

Similar experiments were performed for the actinide Cm(III). Owing to its excellent luminescent properties, a much lower concentration of 3 × 10^−7^ M Cm(III) could be used. As for Eu(III), the Cm(III) TRLFS data can be well explained with a three-species model. One difference between Cm(III) and Eu(III) emission spectra is that not only the intensity or shape of transitions is affected by ligands, but also the peak position itself. The three distinct maxima in the *t* = 0 emission spectra (cf. Figure 3D and Figure S15A, Supplementary Materials) are thus visible evidence for the three-species model. In the pH titration series at pH 2.4, the ^6^D_7/2_ → ^8^S_7/2_ transition of the Cm^3+^ aquo ion was visible as an emission band at 593.8 nm. Upon the increase in pH, a new emission band at 599.4 nm appeared (attributed to Cm(NTA)^0^, lifetime: (100 ± 5) µs), and a further increase in pH yielded a third emission band at 605.5 nm ([Cm(NTA)_2_]^3−^, (263 ± 5) µs). From their respective lifetimes, 5.6 ± 0.3 and 1.6 ± 0.5 remaining water molecules in the first coordination sphere of the Cm^3+^ ion were determined (see Equation (2)).

From the pH- and concentration-dependent species distribution, for the 1:1 and the 1:2 complexes, log *β* values at *I* = 0.1 M NaCl of 11.5 ± 0.2 and 20.5 ± 0.1 for Eu(III) and 11.10 ± 0.02 and 19.50 ± 0.03 for Cm(III) (statistical error given, single measurement only), respectively, were obtained by PARAFAC. Extrapolation to zero ionic strength using SIT yielded 13.4 ± 0.1 and 22.4 ± 0.1 (Eu(III)) and 12.90 ± 0.02 and 21.40 ± 0.03 (Cm(III)), respectively. These data compare well with the literature data.

#### 2.2.2. Eu(III) and Cm(III) Complexation by EDTA

Although complexation of EDTA towards Eu(III) is well-known from the literature [25,37,38,39,40], we included this system as an internal reference system to benchmark our setup.

Analogous to NTA, two series of Eu(III) emission spectra were measured: a pH titration series (pH 1.0–9.0) at tenfold excess in EDTA and a ligand titration series at constant pH = 2.0. The former series showed no spectral changes from pH 3.0 to 9.0. This means that only one complex was formed, confirmed by PARAFAC (corresponding spectra, luminescence decays, and distribution for both series are shown in Figures S16 and S17, Supplementary Materials).

**Figure 4:**
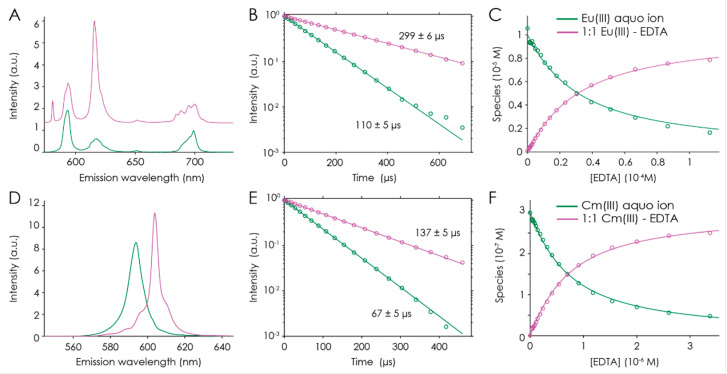
PARAFAC results of TRLFS series of EDTA complexation with Eu(III) (**A**–**C**) and Cm(III) (**D**–**F**). Emission spectra (**A**,**D**), luminescence decays (**B**,**E**), and quantum-yield-corrected PARAFAC distributions (symbols) and corresponding speciation (lines) (**C**,**F**).

PARAFAC analyses of the sum spectra yielded two distinct single-component spectra, one for the Eu^3+^ aquo ion at pH < 2.0, and one for the Eu(III)–EDTA complex at pH ≥ 2.0 Figure 4A–C). The calculated lifetimes of these species are (110 ± 5) and (299 ± 6) µs and, according to Equation (1), corresponding to 9.1 ± 0.5 and 3.0 ± 0.5 water molecules in the metal’s coordination sphere.

The formation constant of Eu(EDTA)^−^ was calculated as log *β* = 17.0 ± 0.1 at the given ionic strength (0.1 M NaCl) by PARAFAC; extrapolation to zero ionic strength using SIT yielded log *β*^0^ = 19.5 ± 0.1. These data are in line with the literature.

Cm(III) complexation by EDTA was studied in an analogous ligand titration series at pH = 2.4. In the presence of EDTA, in addition to the ^6^D_7/2_ → ^8^S_7/2_ transition at 593.8 nm due to the Cm^3+^ aquo ion, a new emission band at 604.0 nm occurred due to the Cm(III)–EDTA complex. Another redshifted peak at 611.0 nm appeared with increasing EDTA concentration due to the vibronic sideband of the organic ligand (see Figure 4D). The observed lifetimes of (67 ± 5) and (137 ± 5) µs, according to Equation (2), correspond to 8.8 ± 0.5 and 3.9 ± 0.5 water molecules in the metal’s coordination sphere. PARAFAC spectral decomposition yielded two distinct species, one of which was the Cm(III) ion, and the other species featured redshift; its prolonged lifetime is attributed to Cm(EDTA)^−^. Based on the speciation obtained at pH 2.4 (Figure 4F), the formation constant of the Cm(III)–EDTA complex was calculated as log *β* = 17.50 ± 0.03 at the given ionic strength (0.1 M NaCl), and extrapolation to zero ionic strength yielded log *β*^0^ = 20.00 ± 0.03. All obtained data (emission maxima, luminescence lifetimes, and complex formation constants) agree very well with those previously published [37,41].

#### 2.2.3. Eu(III) and Cm(III) Complexation by EGTA

**Figure 5:**
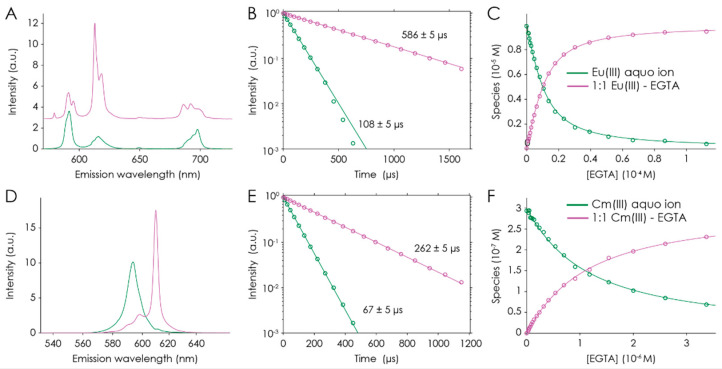
PARAFAC results of TRLFS series of EGTA complexation with Eu(III) (**A**–**C**) and Cm(III) (**D**–**F**). Emission spectra (**A**,**D**), luminescence decays (**B**,**E**), and quantum-yield-corrected PARAFAC distributions (symbols) and corresponding speciation (lines) (**C**,**F**).

Analogous to the Eu(III)–NTA and Eu(III)–EDTA systems, pH and ligand titration series were conducted (see Figures S18 and S19, Supplementary Materials). As of pH 2.8, the spectra remained unaltered, indicating the persistence of the same complex up to pH 9.0. Between pH 2.0 and 2.8, the emission spectra constituted a mixture of the spectra of both the Eu(III) aquo ion and the complex. EGTA-to-Eu(III) titration was performed at constant pH = 3.0. Upon increasing the ligand concentration, the spectra again changed their appearance from purely Eu^3+^ aquo ion-associated to exclusively Eu(III)–EGTA complex-associated. PARAFAC analysis yielded two single-component spectra (Figure 5A), one of which was associated with the Eu^3+^ aquo ion, and one for the EGTA complex. The calculated lifetimes of (108 ± 5) and (586 ± 5) µs along with 9.3 ± 0.5 and 1.2 ± 0.5 coordinating water molecules (Equation (1)), respectively, refer to the Eu^3+^ aquo ion and the Eu(III)–EGTA 1:1 complex. Overall, from both series, it can be concluded that for 10 µM Eu(III), the only complex forming upon octadentate tetraanionic ligand coordination is Eu(EGTA)^−^. Finally, considering the species distribution at pH 3, complex formation constants amounted to log *β* = 17.9 ± 0.2 and log *β*^0^ = 20.5 ± 0.2 at the given ionic strength (0.1 M NaCl) and when extrapolated to zero ionic strength, respectively.

Cm(III) complexation by EGTA was examined by means of the same approach, that is, in dependence on both pH (see Figure S20, Supplementary Materials) and ligand concentration. Up to pH 2.2 or in absence of EGTA, the spectrum indicated the Cm^3+^ aquo ion as the only species. As of pH 3.4 for tenfold excess EGTA, the spectra remained unchanged, indicating the predominance of the same species, viz., the Cm(III)–EGTA complex. Along the ligand titration series (presence of EGTA) as well as in the range 2.4 ≤ pH ≤ 3.2, the spectra were again a molar-fraction-weighted superposition of the occurring species, accompanied by a successive decrease in the intensity of the Cm^3+^ aquo ion’s ^6^D_7/2_ → ^8^S_7/2_ transition at 593.8 nm and the simultaneous increase in intensity of the Cm(III)–EGTA complex’s emission band emerging at 609.0 nm. From both series, two single-component spectra were obtained from PARAFAC (Figure 5D). Along with the calculated lifetimes of (67 ± 5) and (262 ± 5) µs, corresponding to 8.8 ± 0.5 and 1.6 ± 0.5 water molecules in the metal’s coordination sphere (according to Equation (2)), these two species were the Cm^3+^ aquo ion and Cm(EGTA)^−^, respectively. Based upon the species distribution obtained at pH 3 (Figure 5F), the complex formation constants were calculated by PARAFAC as log *β* = 18.60 ± 0.01 (0.1 M NaCl), and upon extrapolation to zero ionic strength using SIT, they were log *β*^0^ = 21.20 ± 0.01, which is in good agreement with the literature.

### 2.3. Isothermal Titration Calorimetry (ITC)

**Figure 6:**
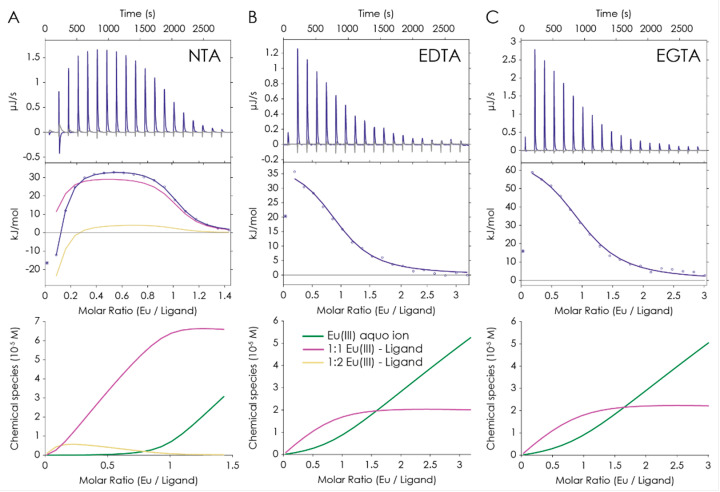
ITC of Eu^3+^ binding to NTA (**A**), EDTA (**B**), and EGTA (**C**). **Top** row: Thermograms obtained from titration of Eu^3+^ to the corresponding ligand. **Middle** row: Integrated heat and best fit. **Bottom** row: Speciation distribution of the measurement. For each ligand, the pictures represent one independent experiment.

ITC was used to determine the thermodynamic parameters (see Figure 6 and Figure S22, Supplementary Materials). The evaluation was conducted according to a procedure described previously [42]. Complex formation constants as well as reaction enthalpy and entropy values were determined for Eu(III) complexation by the three complexones. Table 2 depicts the results obtained from calorimetry and summarizes the determined quantities. Δ*G*^298^ and Δ*S* values were calculated using Equations (3) and (4).

(3) $${\Delta G}^{298}=-RT\ln K$$

(4) $${\Delta G}^{298}=\Delta H-T\Delta S$$

In the case of NTA, measurements showed the formation of both complexes, Eu(NTA)^0^_aq_ and Eu(NTA)_2_^3−^. Logarithmic complex formation constants were determined to be 11.4 ± 0.1 and 20.2 ± 0.2, which is in good agreement with the log *β* values determined with TRLFS. Reaction enthalpy, Δ*H*, decreased from (25.5 ± 1.9) kJ mol^−1^ for the 1:1 complex to −(36.6 ± 3.6) kJ mol^−1^ for the 1:2 complex. The same trend was observed again for the calculated reaction entropy, Δ*S*, which decreased from (113 ± 4) J mol^−1^ K^−1^ to −(91 ± 12) J mol^−1^ K^−1^. The calculated Gibbs energy values at 25 °C, Δ*G*^298^, were −8.1 and −9.5 kJ mol^−1^, respectively. For Eu(EDTA)^−^ and Eu(EGTA)^−^, the measured complex formation constants log *β* were 17.0 ± 0.1 and 18.0 ± 0.2, and the Δ*H* were (39.2 ± 1.1) and (66.7 ± 6.4) kJ mol^−1^, respectively. The calculated Δ*S* were (161 ± 4) and (255 ± 21) J mol^−1^ K^−1^, and the Δ*G*^298^ were −9.1 and −9.2 kJ mol^−1^, respectively. For the 1:1 complexes of all three complexones, the formation reactions with Eu(III) showed an endothermic entropy-driven behavior. In contrast, the formation of the 1:2 Eu(III)–NTA complex was exothermic.

The entropy-driven character of the equimolar complexation reactions is attributed to the ligand’s denticity along with the liberation of water molecules from the metal (four, six, or eight water molecules replaced by one NTA, EDTA, and EGTA, respectively) as well as by stripping water molecules from the ligands’ hydration shell along with breaking hydrogen bonds to the carboxylic and amino groups.

### 2.4. Comparison of the Eu(III) and Cm(III) Complexes of NTA, EDTA, and EGTA

The complex formation of Eu(III) and Cm(III) with the three multidentate ligands NTA, EDTA, and EGTA was investigated with the complementary methods NMR, TRLFS, and ITC. Similarities and differences between the systems were observed.

NTA forms 1:1 and 1:2 complexes with Ln(III) [33]. Luminescence lifetime analyses showed that the Eu(III)–NTA 1:1 complex had a significantly shorter lifetime than the 1:2 complex ((197 ± 0.5) vs. (696 ± 12) µs), in agreement with the reported 190 and 725 µs [36]. The corresponding five and one water molecules remaining in the first coordination sphere indicated that Eu(III)’s coordination number (CN) of nine was also retained in the NTA complexes Eu(NTA)^0^_aq_ and Eu(NTA)_2_^3−^. The NTA ligand coordinates upon tetradentate binding, cf. Equation (1). The crystal structure of K_3_Eu(NTA)_2_ [43], obtained from solutions prepared with a Eu/NTA ratio of 1:2, revealed the Eu(NTA)_2_^3−^ unit matching the spectroscopically derived structure in aqueous medium. The complex formation constants showed a high affinity of NTA towards Eu(III). The log *β*^0^ values of 13.4 ± 0.1 and 22.4 ± 0.1 compare well with the literature data [22] and, according to Anderegg [32], reveal a remarkably large formation constant for a second NTA^3−^ coordinating to Eu(III). The predominance of the complex species strongly depends on the metal/ligand ratio present in the solution (cf. species distribution in Figure S14, Supplementary Materials) rather than on the pH. However, as of pD > 7.0, Eu(NTA)^0^_aq_ could not compete with hydrolysis, whereas Eu(NTA)_2_^3−^ remained stable up to pD 12.0 even in millimolar solution.

NTA complexes of Cm(III) and Eu(III) showed similar behavior. A Cm(NTA)^0^_aq_ and a Cm(NTA)_2_^3−^ complex were observed with lifetimes of (100 ± 5) and (263 ± 5) µs, respectively. The literature reports 11.30 ± 0.01 (*I* = 0.5 M NaClO_4_) [44] and 11.0 (*I* = 0.1 M NaCl) [45] as complex formation constants for the Cm(NTA)^0^ complex, which is in good agreement with the log *β* = 11.10 ± 0.02 found in the present work for *I* = 0.1 M NaCl. For the [Cm(NTA)_2_]^3−^ complex, the literature reports log *β* = 20.13 at *I* = 0.1 M NaCl [45], which is again in good agreement with the 19.50 ± 0.03 found in this work for *I* = 0.1 M NaCl.

Although the complexation of EDTA towards Eu(III) is well known from the literature [25,37,38,39,40], we included this system as an internal reference system to benchmark our setup. Reported and observed results are in good agreement, evidencing that the 1:1 complex is [Eu(EDTA)(H_2_O)_3_]^−^. As expected from the structure, EDTA acted as a hexadentate ligand. Upon replacing water ligands, the changing ligand field translated into significant changes in the Eu(III) emission spectra. Complex formation constants were obtained from both TRLFS and ITC. The log *β* values obtained from independent methods (17.0 ± 0.1 in 0.1 M NaCl) match perfectly, and are in line with those from the literature, viz., 16.69 ± 0.08 [40], 17.35 ± 0.06 [38], and 17.52 ± 0.03 [37]. Like Eu(III), Cm(III) forms a 1:1 complex with EDTA. The observed redshift of the emission maximum (604.0 nm) is in quite good agreement with the literature values (603.9 nm) [41]. In addition, the observed lifetime of (137 ± 5) µs along with the calculated formation constant of log *β* = 17.5 ± 0.03 (0.1 M NaCl) also agrees very well with the previously published (138 ± 5) µs and log *β* = 17.86 ± 0.04 (0.1 M NaClO_4_) [37]. Interestingly, the EDTA complex of Cm(III) is slightly more stable than that of Eu(III).

EGTA formed strong 1:1 complexes with both trivalent europium and curium, in agreement with similar Eu(III) studies [46,47,48]. The excited-state lifetime of this complex is in good agreement with the previously published figures [48]. In this complex, eight donor atoms of EGTA (one oxygen of each of the four carboxylates, two ether-bridge oxygens, and two tertiary amino nitrogens) are bound to the metal center, so EGTA acts as an octadentate ligand. This complexation motif in aqueous solution is in agreement with the structure of [Eu(EGTA)(H_2_O)]^−^ in single crystals [47]. The complex formation constant of the Eu(III)–EGTA complex was determined as log *β* (0.1 M KNO_3_) of 17.1 ± 0.1 using the mercury electrode (pHg method) [49], against the complex stability of the Hg(II)–EGTA complex. Our log *β* (0.1 M NaCl) values, obtained from direct spectroscopic and calorimetric measurements of the formation of the EGTA complexes of Eu(III) as well as Cm(III), yielded 17.9 ± 0.2 and 18.0 ± 0.2 as well as 18.60 ± 0.01, respectively. An alternative titration series at elevated pH (pH 6.0) demonstrated the very high affinity of EGTA towards Eu(III) even at quite low ligand concentrations (Figure S21, Supplementary Materials). This fast saturation is not suitable for the direct determination of complex formation constants as it does not allow for the correct measurement of the equilibrium free metal concentration required for the law of mass action. In contrast, when reducing the ligand’s affinity upon increasing the H^+^ concentration, i.e., adjusting it to sufficiently low pH, the concentration-dependent behavior is asymptotic, enabling correct calculations. Consequently, this offers direct accessibility to high-affinity ligand complexes, as demonstrated by the used approach.

Overall, it appears that, independent of the ligand, the Cm(III) complexes show higher complex formation constants than the corresponding Eu(III) complexes (cf. Table 3). Also, the complex formation constants of the 1:1 complexes increase in the order NTA < EDTA < EGTA for both Eu(III) and Cm(III). The complex formation constants, therefore, depend on the denticity of the ligand. The complex formation constants of the Eu(III) complexes were determined by independent methods working with varying concentrations. The determined log *β* values are in quite good agreement among one another and show the validity over wide concentration ranges. Therefore, the presented workflow and mixture of complementary methods is a good choice to investigate such systems.

## 3. Materials and Methods

### 3.1. Starting Material and Stock Solutions

*Caution! Curium is a radioactive element of high radiotoxicity requiring special precautions for handling radioactive materials, and all studies were conducted in a laboratory dedicated to actinide research.*

All chemicals were used as obtained. Stock solutions were prepared by weighing and dissolving appropriate amounts of EuCl_3_·6H_2_O (99.99%, Sigma-Aldrich, Taufkirchen, Germany), Na_2_HNTA (≥99%, Sigma-Aldrich), H_4_EDTA (≥99%, Roth, Karlsruhe, Germany), and H_4_EGTA (≥99%, Roth) in NaCl (99.5%, Roth) containing Milli-Q H_2_O (18.2 MΩ cm, Millipore, Merck, Darmstadt, Germany) and D_2_O (99.98% D, Deutero, Kastellaun, Germany) aqueous solutions. pH was adjusted with HCl (1.0 M, 0.1 M, and 0.01 M) and NaOH (1.0 M, 0.1 M, and 0.01 M) in D_2_O solutions, likewise, DCl and NaOD (both >99% D, Deutero), using a pH meter (inoLab pH 730, Xylem, Weilheim, Germany) equipped with a pH electrode (SCHOTT, BlueLine, SI Analytics, Mainz, Germany).

^248^Cm was obtained from the transplutonium element production facilities at Oak Ridge National Laboratory, Oak Ridge, TN, USA. Appropriate dilutions were made from a 295 µM Cm(ClO_4_)_3_ stock solution.

### 3.2. Sample Preparation

#### 3.2.1. NMR pH Titration Series for p*K*_a_ Determination

The obtained 40 mM NTA (1 M NaCl), as well as 1 mM solutions of NTA, EDTA, and EGTA, respectively, containing 0.1 M NaCl, were prepared in 10% D_2_O in Milli-Q H_2_O, in the pH range 0.6–12.0, with increments of 0.2 to 0.4 pH units. pH was corrected for deuterium according to the common pD = pH(read) + 0.4 in pure D_2_O. Since the reading of the pH meter is very nearly a linear function of the atom-% deuterium [56], the used 10% D_2_O contents afforded for addition of 0.04 pH units.

#### 3.2.2. Eu(III) Complexation by NTA and EGTA Studied by NMR

For NTA studies, solutions were prepared in D_2_O containing 1 M NaCl and either 15 mM Eu(III) and 30 mM NTA at pD 1.0–12.0 or 18 mM Eu(III) and 18 mM NTA at pD 3.0–12.0. In the case of the equimolar series, samples above pD 7.0 showed formation of a white solid. Therefore, these solutions were centrifuged and the obtained clear supernatants were investigated. In solutions with excess NTA, no precipitation occurred. For the EGTA series, samples 30 mM each in EGTA and Eu(III) were prepared analogously and adjusted to pD values of 1.0, 3.0, 5.0, 7.0, and 9.0, without formation of precipitates.

#### 3.2.3. Eu(III) and Cm(III) Complexation by NTA, EDTA, and EGTA Studied via TRLFS

For Eu(III) studies, all samples were prepared in Milli-Q H_2_O containing 10 µM Eu(III) and 0.1 M NaCl. For ligand titration series (in triplicates per ligand), ligand concentration was varied between 0 and 112.5 µM, at constant pH values, i.e., pH = 6.0 for NTA, pH = 2.0 for EDTA, and pH = 3.0 for EGTA. For pH titration series, pH was varied between 1.0 and 9.0, at constant ligand concentration of 1 mM for NTA as well as 100 µM for EDTA and EGTA. Similar experiments have been conducted with 0.3 µM Cm(III) in 0.1 M NaCl aqueous solution, with ligand concentration titrated from 0 through 450 µM at pH = 5.0 for NTA, and from 0 through 3.37 µM at pH = 2.4 for EDTA and pH = 3.0 for EGTA, and pH titrated between 1.0 and 9.0 in presence of either 30 µM NTA or 3 µM EGTA.

#### 3.2.4. Eu(III) Complexation by NTA, EDTA, and EGTA Studied via ITC

All experiments were performed in 0.1 M NaCl Milli-Q H_2_O solution. Titrations were performed with 600 µM Eu(III) and 90 µM NTA at pH = 6.0, and 450 µM Eu(III) and 30 µM EDTA at pH = 2.3, as well as 30 µM EGTA at pH = 3.0.

### 3.3. NMR Spectroscopy

NMR spectra were recorded at 25 °C with an Agilent DD2-600 NMR system, operating at 14.1 T with corresponding ^1^H and ^13^C resonance frequencies of 599.8 and 150.8 MHz, respectively, using a 5 mm oneNMR™ probe. ^1^H NMR spectra were measured by accumulating 32–128 scans, depending on concentrations and line widths, using 2 s of acquisition time and relaxation delay, respectively, applying a 2 s pre-saturation pulse on the water resonance for water signal suppression. For ^13^C NMR measurements, 1024 scans were accumulated upon applying 4 s relaxation delay after a 3.3 µs excitation (30°) pulse and 1 s acquisition time, with ^1^H broadband decoupling.

For Eu(III)–EGTA complex signal assignment, heteronuclear single-quantum coherence (HSQC) and heteronuclear multiple-bond correlation was performed using sequences with gradient selection and adiabatic pulses. H,C-HSQC and H,C-HMBC spectra of the pD 5.0 sample 30 mM in each Eu(III) and EGTA were obtained at 70 °C on an Agilent MR-400 NMR system (9.4 T, 399.8 MHz ^1^H and 100.8 MHz ^13^C resonance frequencies, 5 mm oneNMR™ probe), acquired with 4k × 1k complex points in *F*_2_ and *F*_1_, 64 and 100 transitions per *F*_1_ increment, and a relaxation delay of 1 s, respectively. For polarization transfer, (2*J*)^−1^ delays of 3.85 and 100 ms were opted, corresponding to 130 Hz ^1^*J* in HSQC and 5 Hz ^n^*J* in HMBC, respectively.

### 3.4. Luminescence Spectroscopy

Solutions were stirred in 10 mm path length Hellma Analytics 4 mL quartz cells. The cuvette was placed in a cuvette holder which was connected via a light guide to a spectrograph (Andor, Belfast, UK, SR-303i-A). For recording the spectra, an ICCD (Andor iStar, DH320T-18U-63) was used. The excitation wavelength (Ekspla, Vilnius, Lithuania, NT230, ~5 ns pulse) was 394 nm (grating: 300 mm^−1^). For Cm(III) luminescence spectroscopy, a unique pulsed flash lamp pumped Nd:YAG OPO laser system (Powerlite Precision II 9020 laser equipped with a Green PANTHER EX OPO from Continuum, Santa Clara, CA, USA) was used. The laser system was equipped with a delay generator (Stanford Research Systems Inc., Sunnyvale, CA, USA, Model DG535). The luminescence spectra were detected using an optical multi-channel analyzer system, consisting of an Andor Kymera 328i monochromator and spectrograph with a grating of 150, 300, 600, and 1200 lines per mm (Oxford Instruments, Abingdon, UK) and an Andor iStar ICCD camera (ICCD 05933, Andor). The excitation wavelength was 396 nm (grating: 300 mm^−1^).

### 3.5. Calorimetry

ITC titrations were performed in triplicates along with control experiments (Eu(III) titration to 0.1 M NaCl) on a Malvern Panalytical, Kassel, Germany, MicroCal PEAQ-ITC instrument.

The ligand was loaded in the cell, Eu(III) in the syringe, and we added in 19 steps; the first was 0.2 µL and the following were 2.0 µL each in volume. Between injections, the system was equilibrated for 150 s. The reference cell was filled with water, the stirring speed set to 750 rpm, and the initial delay was set to 60 s.

For an accurate thermodynamic characterization of the complexes, it is useful to avoid any background reactions. As has been shown recently, many buffers show some interaction with lanthanides [57]. Therefore, work was carried out in unbuffered solutions. For EDTA and EGTA, due to the low pH values, no pH change was observed during the titration. The situation is different for NTA. The pH of the solution is lowered by the protons released during complexation. This gradual pH change was taken into account in the evaluation of the data.

### 3.6. DFT Calculation

DFT calculations on Eu(III)–NTA 2:2 complex was performed using Gaussian 16 program (Gaussian Inc., Wallingford, CT, USA) [58]. Geometries were optimized in the aqueous phase (eps = 78.3553) at the B3LYP level using the PCM solvation model with UFF (universal force field) radii. The large core effective core potential (LC-ECP) as well as the corresponding basis sets suggested by Dolg et al. [59] were used on Eu. For N, O, C, and H, all-electron valence triple-ζ basis set plus polarization and diffuse functions were used [60]. The LC-ECP used in this study is specifically designed for the trivalent Eu incorporating six unpaired 4f electrons into the core potential, thereby enabling “closed-shell” calculations on Eu^3+^, which has the formal electronic configuration of the septet state. This simplification is justified because the 4f orbital is strongly contracted, shielded by valence orbitals, and does not actively participate in the chemical bond between Eu and NTA. We have successfully used the same methodology in our previous publications [61,62]. The final geometries were confirmed to be the energy minimum through vibrational frequency analysis where no imaginary frequency was found to be present.

### 3.7. Data Processing Software

For calculating complex formation constants extrapolated to zero ionic strength by means of SIT, the program Aqua Solution Software (Acadsoft, York, UK) [63] was used. NMR spectra were processed with MestReNova, version 6.0.2., Mestrelab Research S.L., Santiago de Compostela, Spain [64]. The PARAFAC-assisted evaluation of luminescence spectra is described elsewhere [35]. Creation of graphs for numerical data visualization and data fitting by non-linear sigmoidal dose–response fit algorithm was performed with Origin 2019, version 9.6.0.172, OriginLab Corporation, Northampton, MA, USA. The DFT-calculated structure was visualized with Avogadro [65].

## 4. Conclusions

Demonstrating thermodynamic studies on the Eu(III) and Cm(III) interaction with complexones, the approach of this work is commonly applicable to many other metal–ligand systems. This workflow offers direct access to complex formation constants, especially for high-affinity systems, by using complementary spectroscopic and calorimetric methods. Therefore, it provides a clear advantage over indirect approaches such as metal ion competition experiments or potentiometric methods.

The three investigated complexones form 1:1 complexes with both Eu(III) and Cm(III). Besides the established Eu(III)–NTA 1:1 and 1:2 complexes, we observed, for the first time, the existence of a Eu(III)–NTA 2:2 complex of millimolar metal and ligand concentrations.

The gained data, especially the log *β* values, show the close relationship between Eu(III) and Cm(III). The Cm(III) complexes appear to have slightly higher complex formation constants, most probably based on the higher percentage of covalency in actinide bonding. Nevertheless, the complex stabilities for both metals are in the same order of magnitude. This corroborates the often-referred similarity between lanthanides and actinides. Therefore, the approach to use Eu(III) as an analogue of An(III) can be used with few exceptions. Based on the high stabilities of the complexes, on the one hand, complexones from the (poly)aminopolycarboxylate family are—at least from a thermodynamic point of view—suitable as both decontamination and decorporation agents. On the other hand, if (accidentally) released, for a broad concentration and pH range, this kind of compound carries the risk to mobilize and spread (radio)toxic metals. Such information is crucial for a reliable long-term safety assessment of nuclear waste repositories, as a (re-)mobilization of radionuclides during ground water ingress is to be avoided.

Based on the obtained data, EGTA appears to be well suited as a decorporation agent, especially for trivalent actinides and lanthanides, because the resulting complexes are readily water soluble and therefore can be excreted, e.g., in urine.

## Figures and Tables

**Table 1:** p*K*_a_ values of NTA, EDTA, and EGTA determined in the present work (p. w.) and from the literature.

	p*K*_a_				
	1	2	3	4	
*NTA*	NTA^0^ → NTA^−^	NTA^−^ → NTA^2−^	NTA^2−^ → NTA^3−^		
*I* = 0.1 M NaCl	1.50 ± 0.18	2.67 ± 0.22	9.58 ± 0.02	—	p. w.
*I* → 0	1.71 ± 0.18	3.09 ± 0.22	11.45 ± 0.02	—	p. w.
*I* = 0.1 M KCl	1.5	2.52	9.59	—	[26]
*I* = 0.5 M KNO_3_	1.57 ± 0.06	2.64 ± 0.04	9.57 ± 0.06	—	[27]
*EDTA*	EDTA^0^ → EDTA^−^	EDTA^−^ → EDTA^2−^	EDTA^2−^ → EDTA^3−^	EDTA^3−^ → EDTA^4−^	
*I* = 0.1 M NaCl	1.12 ± 0.06	2.50 ± 0.02	6.10 ± 0.01	9.65 ± 0.01	p. w.
*I* → 0	1.36 ± 0.06	2.96 ± 0.02	6.74 ± 0.01	10.45 ± 0.01	p. w.
*I* = 0.1 M KCl	2.0	2.67	6.16	10.26	[28]
*I* = 0.5 M KNO_3_	1.89 ± 0.06	2.68 ± 0.04	6.26 ± 0.06	10.50 ± 0.02	[27]
*EGTA*	EGTA^0^ → EGTA^−^	EGTA^−^ → EGTA^2−^	EGTA^2−^ → EGTA^3−^	EGTA^3−^ → EGTA^4−^	
*I* = 0.1 M NaCl	1.45 ± 0.04	2.28 ± 0.08	9.25 ± 0.01	9.25 ± 0.01	p. w.
*I* → 0	1.69 ± 0.04	2.74 ± 0.08	9.89 ± 0.01	10.05 ± 0.01	p. w.
*I* = 0.1 M KCl	2.0	2.65	8.85	9.46	[28]

**Table 2:** Thermodynamic data for Eu(III) complexation by NTA, EDTA, and EGTA, obtained from isothermal titration calorimetry at 25 °C in aqueous solutions containing 0.1 M NaCl.

	Eu(NTA)^0^_aq_	Eu(NTA)_2_^3−^	Eu(EDTA)^−^	Eu(EGTA)^−^
Δ*H* (kJ mol^−1^)	25.5 ± 1.9	−(36.6 ± 3.6)	39.2 ± 1.1	66.7 ± 6.4
Δ*S* (J mol^−1^ K^−1^)	113 ± 4	−(91 ± 12)	161 ± 4	255 ± 21
Δ*G*^298^ (kJ mol^−1^)	−8.1	−9.5	−9.1	−9.2

**Table 3:** Formation constants of Eu(III) and Cm(III) complexes with NTA, EDTA, and EGTA.

Species	log *β* ^a^ (TRLFS)	log *β*^0 b^ (TRLFS)	log *β* ^a^ (ITC)	log *β*^0 b^ (ITC)	log *β* (Literature)
Eu^3+^ + NTA^3−^ → [Eu(NTA)]^0^_aq_	11.5 ± 0.2	13.4 ± 0.1	11.4 ± 0.1	13.3 ± 0.1	11.1 ± 0.1 [27] 13.23 ^b^ [22]
Cm^3+^ + NTA^3−^ → [Cm(NTA)]^0^_aq_	11.10 ± 0.02 ^c^	12.90 ± 0.02 ^c^	—	—	11.00 ^a^ [45] 11.30 ± 0.01 ^d^ [44]
[Eu(NTA)]^0^_aq_ + NTA^3−^ → [Eu(NTA)_2_]^3−^	20.5 ± 0.1	22.4 ± 0.1	20.2 ± 0.2	22.1 ± 0.2	20.42 ^a^ [45]
[Cm(NTA)]^0^_aq_ + NTA^3−^ → [Cm(NTA)_2_]^3−^	19.50 ± 0.03 ^c^	21.40 ± 0.03 ^c^	—	—	20.13 ^a^ [45]
Eu^3+^ + EDTA^3−^ → [Eu(EDTA)]^0^_aq_	17.0 ± 0.1	19.5 ± 0.1	17.0 ± 0.1	19.6 ± 0.1	16.2 ± 0.1 [27] 16.69 ± 0.08 ^h^ [40] 17.35 ± 0.06 ^e^ [38] 17.52 ± 0.03 ^f^ [37]
Cm^3+^ + EDTA^3−^ → [Cm(EDTA)]^0^_aq_	17.50 ± 0.03 ^c^	20.00 ± 0.03 ^c^	—	—	16.9 ± 0.1 [50] 17.10 ^a^ [45] 17.86 ± 0.04 ^f^ [37] 18.45 ^g^ [51]
Eu^3+^ + EGTA^3−^ → [Eu(EGTA)]^0^_aq_	17.9 ± 0.2	20.5 ± 0.2	18.0 ± 0.2	20.5 ± 0.2	17.1 ± 0.1 ^e^ [49] 17.65 ± 0.08 ^f^ [52] 17.8 ^e^ [49]
Cm^3+^ + EGTA^3−^ → [Cm(EGTA)]^0^_aq_	18.60 ± 0.01 ^c^	21.20 ± 0.01 ^c^	—	—	17.94 ± 0.09 ^f^ [52]

^a^ *I* = 0.1 M (NaCl). Errors are standard deviation of at least three independent experiments. ^b^ *I* extrapolated to zero ionic strength. The SIT ion interaction coefficients used for the calculations were 0.26 ± 0.01 for Eu(III) in NaCl [53]. For Cm(III), no data were available, and Am(III) coefficients (Am(III) in NaCl: 0.23 ± 0.02 [54]) were used as substitute. For EDTA^4−^ and EGTA^4−^, the data for EDTA (H_3_EDTA^−^: −0.33 ± 0.14, H_2_EDTA^2−^: −0.37 ± 0.14, HEDTA^3−^: −0.10 ± 0.14, EDTA^4−^: 0.32 ± 0.14, all in NaCl [54]) were used. As substitute for NTA, the SIT coefficients for propanetricarboxylic acid, H_3_PTC (−0.01 ± 0.04 for H_2_PTC^−^, 0.06 ± 0.04 for HPTC^2−^ and 0.11 ± 0.04 for PTC^3−^, each in NaCl [55]), were used. ^c^ The given error is the fitting error of a single measurement. ^d^ *I* = 0.5 M (NaClO_4_). ^e^ *I* = 0.1 M (KNO_3_). ^f^ *I* = 0.1 M (NaClO_4_). ^g^ *I* = 0.1 M (NH_4_ClO_4_). ^h^ *I* = 0.09 M (KCl).

## Data Availability

All data are included in this paper.

## Supplementary Materials

The following supporting information can be downloaded at: https://www.mdpi.com/article/10.3390/molecules28124881/s1, Figures S1 and S2: ^1^H NMR pH titration spectra of 40 mM NTA and corresponding p*K*_a_ determination; Figure S3: Structures of all considered ligand species; Figure S4: ^1^H and ^13^C NMR single-component spectra of the Eu(III)–NTA 1:1, 1:2, and 2:2 complexes; Figures S5 and S6: pD-dependent ^1^H and ^13^C NMR spectra of the Eu(III)–EGTA complex and signal assignment by one- and two-dimensional NMR techniques; Figure S7: DFT-calculated structure of the Eu(III)–NTA 2:2 complex; Figures S8–S11: Structures of all other considered complexes; Figures S12–S21: PARAFAC results of Eu(III) and Cm(III) TRLFS series for NTA, EDTA, and EGTA, in dependence of pH and ligand concentration; Figure S22: ITC results of Eu(III) complexation by NTA, EDTA, and EGTA; Tables S1 and S2: Complex formation constants and thermodynamic parameters for Eu(III) complexation by NTA, EDTA, and EGTA, obtained from TRLFS and ITC measurements. Reference [46] is also cited in the Supplementary Materials.

## Sample Availability

Samples of the compounds are not available from the authors.
