# Supplementary material for: Eu(III) and Cm(III) Complexation by the Aminocarboxylates NTA, EDTA, and EGTA Studied with NMR, TRLFS, and ITC—An Improved Approach to More Robust Thermodynamics
Source: Molecules. 2023 Jun 20;28(12):4881. doi: 10.3390/molecules28124881 (PMC10304629; doi:10.3390/molecules28124881)
Supplement: Supplementary file 1 [file molecules-28-04881-s001.zip › molecules-2440971-supplementary.pdf]

Supplementary Materials

Eu(III) and Cm(III) Complexation by the Aminocarboxylates NTA, EDTA, and EGTA Studied with NMR, TRLFS, and ITC – An Improved Approach to More Robust Thermodynamics

Sebastian Friedrich [1,†], Claudia Sieber [1,†], Björn Drobot [1], Satoru Tsushima [1,2], Astrid Barkleit [1], Katja Schmeide [1], Thorsten Stumpf [1], Jerome Kretzschmar [1,*]

[1] Helmholtz-Zentrum Dresden–Rossendorf, Institute of Resource Ecology, 01328 Dresden, Germany.

[2] International Research Frontiers Initiative, Institute of Innovative Research, Tokyo Institute of Technology, Meguro, Tokyo 152-8550, Japan.

*Correspondence: j.kretzschmar@hzdr.de

[†] These authors contributed equally to this work.

pH-titration NMR spectra

NTA

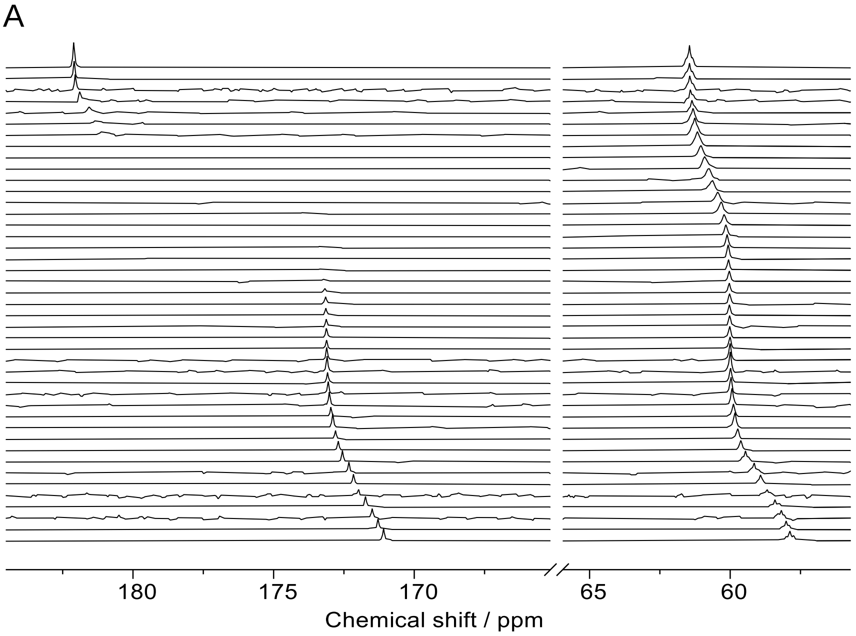

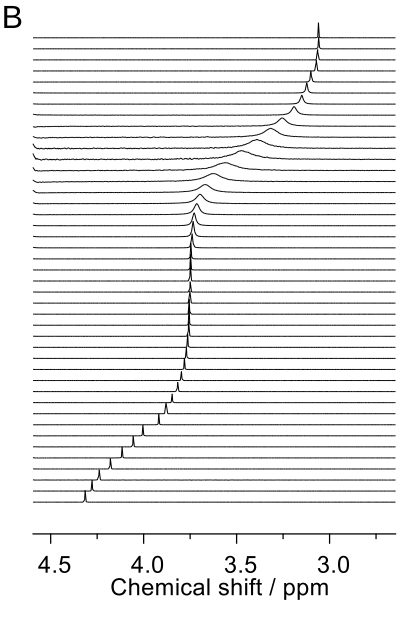

Figure S1. NMR pH-titration series of NTA in the pH range 0.6 – 12.0; pH values increase from bottom to top with increments of 0.2 to 0.4 pH units. (**A**) $^{13}C\{^1H\}$ and (**B**) ^1H NMR spectra obtained from 40 mM NTA in 1 M NaCl aqueous solutions containing 10% D_2O. For clarity, only spectral regions of interest are shown. Note that between pH 6 and pH 9, owing to protonation/deprotonation reactions at the amine nitrogen, NTA's carboxyl ^{13}C signals are broadened to such an extent that the signals cannot be observed.

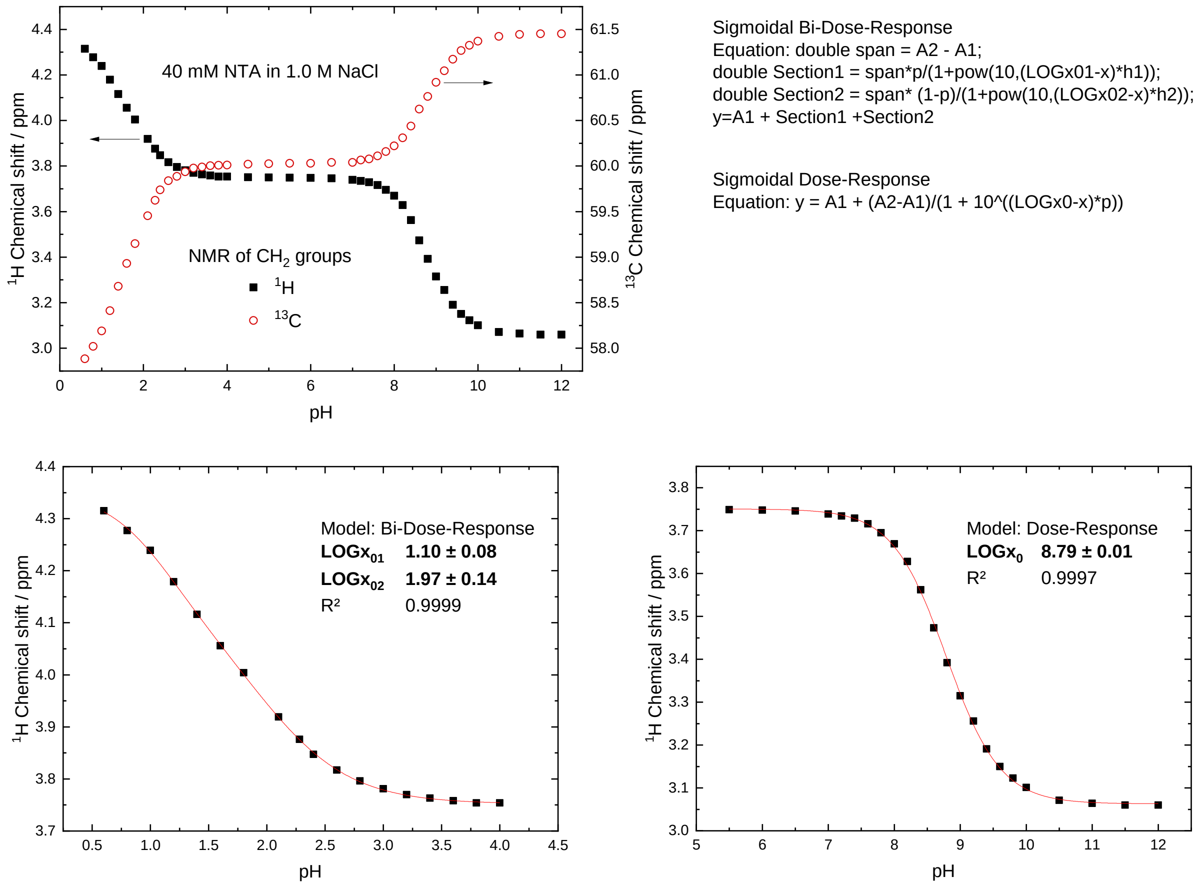

Figure S2. pH-dependent ^{1}H and ^{13}C (CH_2) chemical shift values of 40 mM NTA in 1.0 M aqueous NaCl solution (top) corresponding to the spectra shown in **Figure S1** A and B, along with sigmoidal dose–response fits for determining pK_a values from ^{1}H data. ^{13}C NMR data of the methylene carbon signal reveal similar pK_a values (not shown). ^{13}C NMR data of the carboxyl carbon signal is not applicable upon its intermediate disappearance (**Figure S1** A).

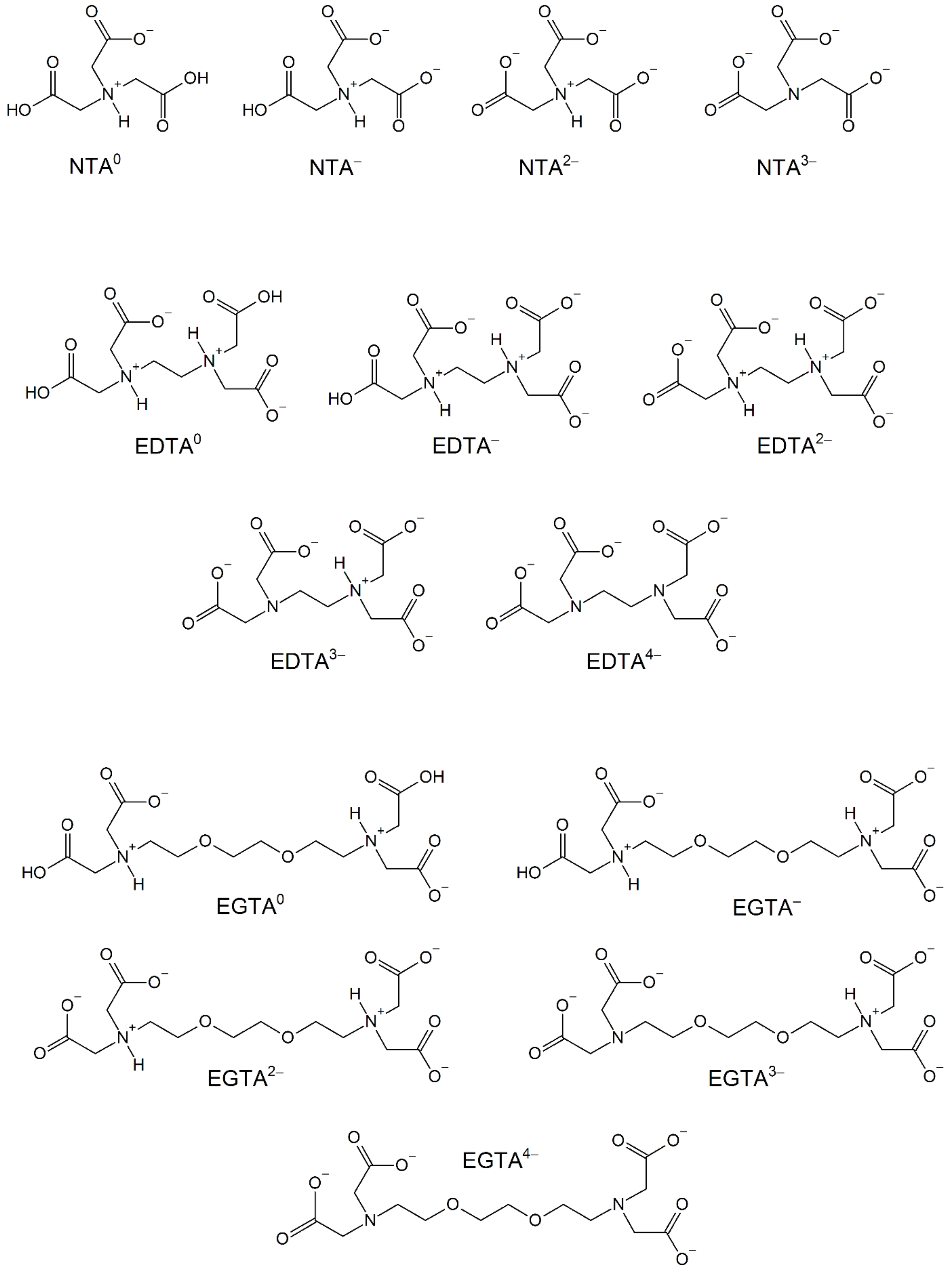

Figure S3. Generic structures of the ligand species considered.

Eu(III) complexation: NMR spectra

NTA

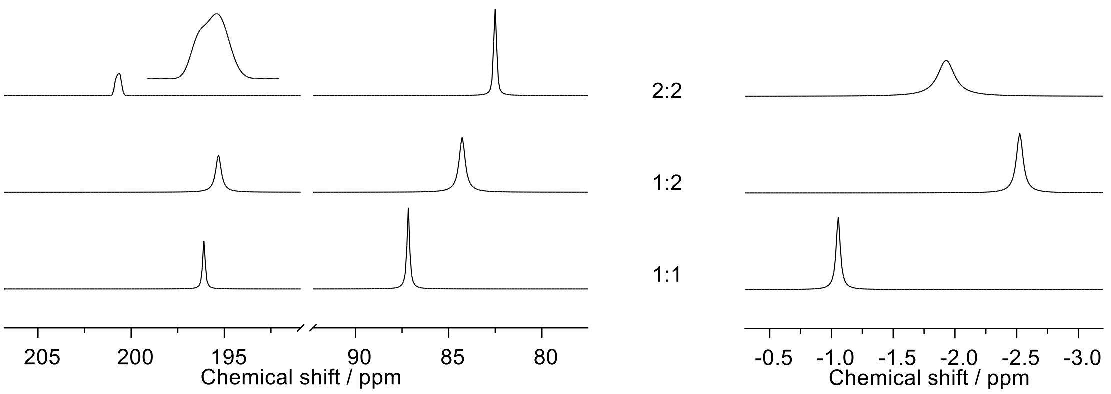

Figure S4. Single-component $^{13}C\{^{1}H\}$ (left) and ^{1}H NMR spectra (right) of the three distinct Eu(III)–NTA complexes obtained from Lorentzian/Gaussian deconvolution. A magnification of the 2:2 complex's carboxyl signals is given as insert.

EGTA

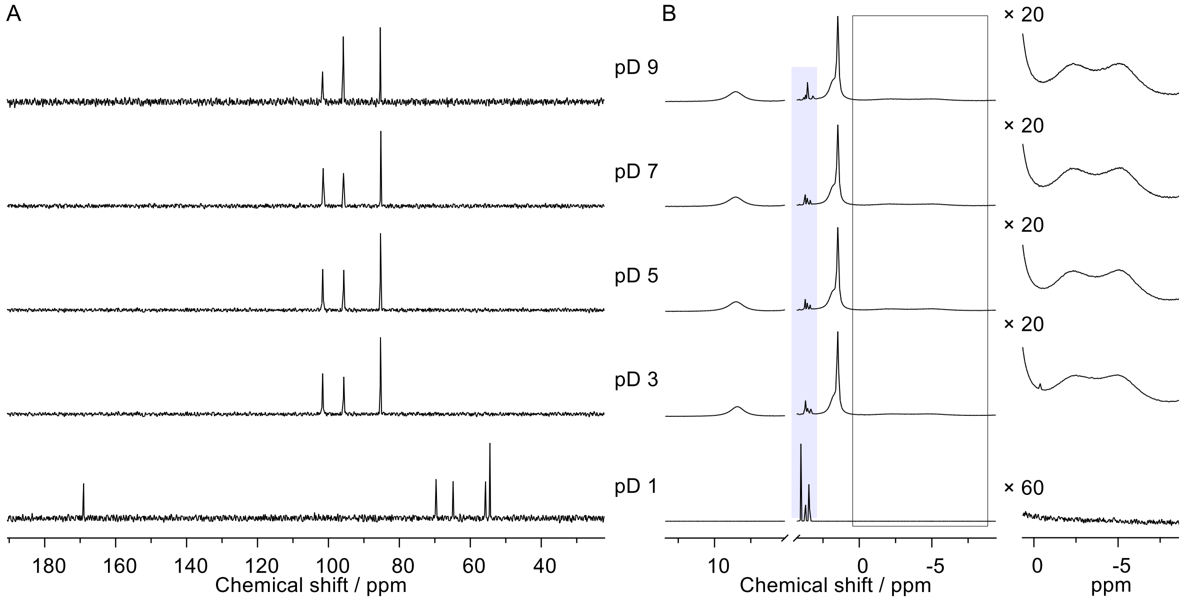

Figure S5. ^{13}C (**A**) and ^{1}H (**B**) NMR spectra obtained from D_2O solutions 30 mM each in EGTA and Eu(III) in dependence on pD at T = 25 °C at 14.1 T (600/150 MHz $^{1}H/^{13}C$). For clarity, only spectral regions of interest are shown. The insert on the right shows magnifications of the spectral region indicated in (**B**). A very small fraction of unbound EGTA can be detected only by ^{1}H NMR (shaded signals in **B**).

Owing to Eu(III)'s paramagnetic effects, in addition to signals being shifted, some are broadened to such an extent that they cannot be observed. Therefore, only three of five ^{13}C signals can be detected; *viz*. those attributed to the complex' N–CH_2–CH_2–O–CH_2 moiety (cf. **Figure S5**), whereas those of the CH_2–COO residues cannot be detected under the given conditions.

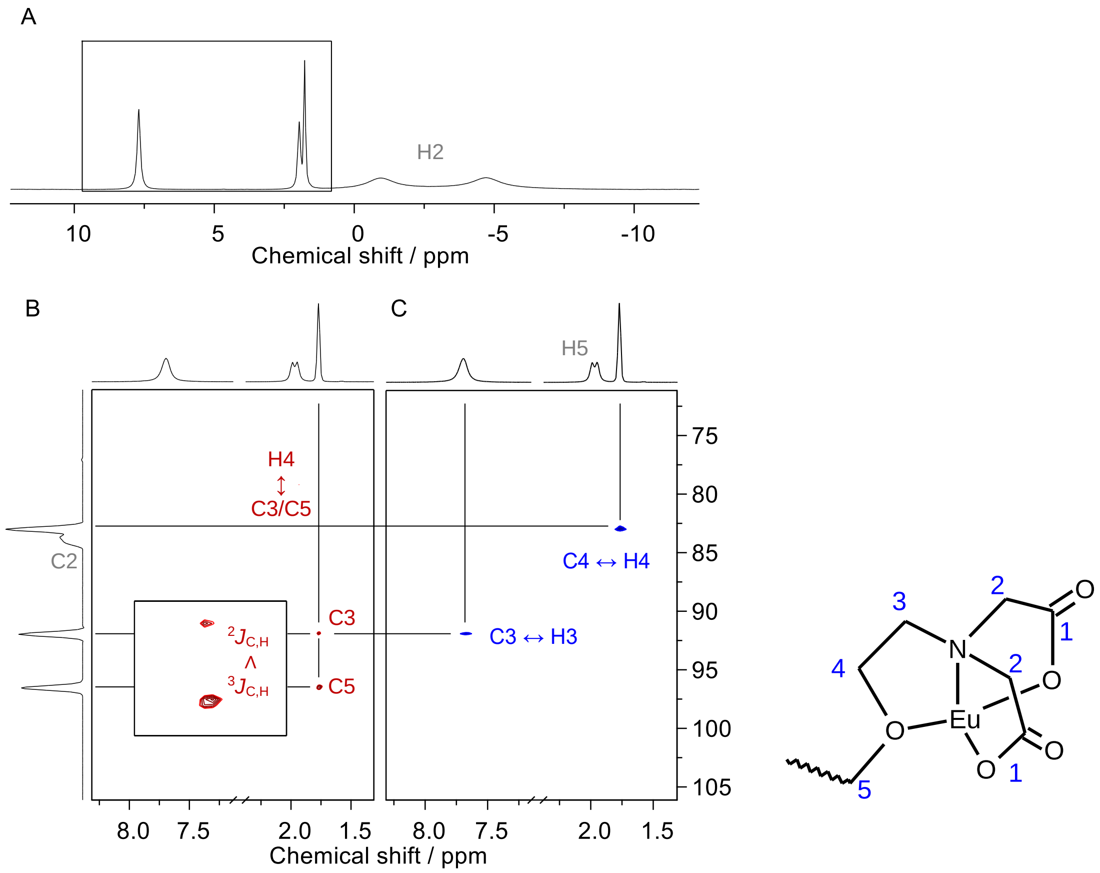

Figure S6. ^{1}H NMR (**A**), H,C-HMBC (8 Hz) (**B**) and H,C-HSQC (130 Hz) (**C**) spectra of pD 5 D_2O solutions 30 mM each in EGTA and Eu(III) obtained at T = 70 °C at 9.4 T (400/100 MHz ^{1}H/^{13}C). An exponential line broadening factor of 20 Hz was applied to the ^{1}H spectrum in **A** to better visualize the two broad high-field signals. The multiple-bond (**B**) and single-bond (**C**) correlation spectra refer to C–H correlations detected for the sharp signals within the indicated region. The insert in **B** is a magnification of the two correlation signals. Shown on the right is a structure fragment of the Eu(III)–EGTA complex for demonstrating the observed correlations respectively stated with the spectra.

Owing to Eu(III)'s effective paramagnetic-induced relaxation (along with intramolecular dynamics [1]), signals can be extremely broadened. Since the carboxyl signal is not observable, and of the remaining four signals one is very broad and three are moderately broad, we assign the former to the iminoacetate methylene group C2, adjacent to the carboxyl, and the latter three to C3, C4, and C5. Correspondingly, the two very broad ^{1}H signals at high field are assigned to the iminoacetate protons (H2), hence the remaining ^{1}H signals belong to methylene protons H3, H4, and H5. Of these, only one methylene group shows multiple-bond correlations to other carbons. Therefore, the carbon these protons are attached to is adjacent to both other carbons, hence C4, and the remaining carbons are C3 and C5. The differences in signal intensity of the multiple-bond correlations (see insert in **B**), is attributed to the respective strengths of $J_{C,H}$ couplings; i.e., in general scalar C–H couplings *via* three bonds are stronger than those *via* two bonds ($^{3}J_{C,H} > {}^{2}J_{C,H}$), and the HMBC acquisition parameters were opted for 8 Hz couplings thus pronouncing the $^{3}J_{C,H}$. Therefore, the weaker coupling refers to C3, and the stronger to C5. From the HSQC spectrum (**C**) methylene protons H4 and H3 can be directly assigned; the remaining ^{1}H signal hence is due to H5. Although partly very broad, the ^{1}H signal integrals in **A** fairly agree with a 4:4:4:*4:4* ratio. That indicates that not all eight but pairs of four iminoacetate protons are respectively equivalent.
Overall, our assignments are in some cases contradictory to those of Aime et al. [1], who assigned the iminoacetate methylene protons to the signal we denoted H5, considering neither the signal integrals, nor their observation of pairwise appearance of corresponding iminoacetate methylene and carboxyl signals (at low temperature). Also, their assignment of C3 and C4 is opposite.

DFT-calculated molecular structure of the Eu(III)–NTA 2:2 complex $[Eu_2(NTA)_2]^0_{aq}$

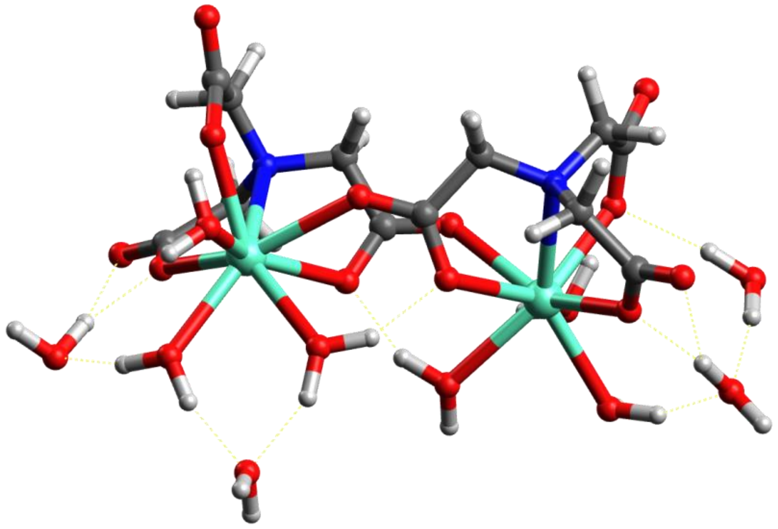

Figure S7. DFT-calculated structure of the Eu(III)–NTA 2:2 complex.

Experiment showed that dimer formation is only triggered above a certain concentration threshold. We assume that the reaction is entropy-driven because of the liberation of water molecules upon dimerization. Interestingly, the energy of the 2:2 complex is lower (more stable) than the double of 1:1 complex. Essentially, formation of the 2:2 complex is definitely possible from the energetics' point of view.

For visualization, cartesian x,y,z coordinates are given.

Eu	2.9276	0.4003	0.4677
Eu	-2.624	0.4211	-0.5799
O	3.5953	0.8187	2.8530
O	-2.9740	-0.3323	-3.0129
O	-3.9818	1.9940	-1.9283
O	-0.9364	1.8486	-1.6848
O	-6.4545	-0.6625	-1.6182
O	-6.2654	1.9939	-0.5793
O	2.5684	4.7059	0.4431
O	4.1997	2.4847	0.3881
O	1.4194	2.2859	0.8910
O	6.3704	2.0826	-1.1114
O	1.0849	0.1101	-1.2131
O	-0.7671	-1.1400	-1.1740
O	3.9771	-3.6764	2.0182
O	5.3794	-0.8288	-3.1246
O	3.6964	-1.4857	1.6306
O	4.5370	0.0967	-1.2673
O	0.8731	-0.4524	1.7033
O	-0.8644	0.8095	1.0765
O	-4.2233	-3.7087	-0.1721
O	-5.2985	1.1723	2.9538

O	-3.7739	-1.5986	-0.7829
O	-4.2861	1.1258	0.9566
N	2.7626	-1.9547	-0.9344
N	-2.7177	-0.9954	1.7275
C	1.3282	-2.2671	-0.9908
C	0.4802	-1.0065	-1.1573
C	3.5342	-2.9966	-0.2245
C	3.3177	-1.6978	-2.2767
C	3.7350	-2.7292	1.2782
C	4.5325	-0.7618	-2.2420
C	-1.3178	-1.3496	2.0186
C	-0.3534	-0.2417	1.5866
C	-3.5785	-2.1863	1.5452
C	-3.2673	-0.0820	2.7519
C	-3.8649	-2.5687	0.0823
C	-4.4014	0.7984	2.2112
H	3.9338	-0.0557	3.1018
H	4.2627	1.4822	3.0606
H	-3.3457	-1.2088	-2.8254
H	-2.1990	-0.4617	-3.5722
H	-4.0634	1.6124	-2.8119
H	-4.8896	2.1808	-1.5854
H	-1.0091	2.3452	-2.5057
H	-0.0650	1.3912	-1.6470
H	-5.6110	-1.0367	-1.3236
H	-6.5047	0.2293	-1.2352
H	-5.7233	1.8040	0.2228
H	-6.9053	2.6849	-0.3814
H	2.4529	5.2068	-0.3726
H	2.6271	5.3413	1.1659
H	3.7779	3.3653	0.4012
H	5.0475	2.5207	-0.1186
H	1.5516	3.2348	0.7130
H	0.4752	2.0429	0.9469
H	5.8821	1.2642	-1.3574
H	7.1345	1.8068	-0.5932
H	1.0272	-2.7361	-0.0523
H	1.0792	-2.9789	-1.7889
H	4.5396	-3.0345	-0.6536
H	3.1011	-3.9917	-0.3653
H	2.5608	-1.1946	-2.8823
H	3.5816	-2.6262	-2.7944
H	-1.0519	-2.2465	1.4573
H	-1.1537	-1.5831	3.0784
H	-4.5576	-1.9821	1.9874
H	-3.1820	-3.0573	2.0733
H	-2.4763	0.5978	3.0761
H	-3.6130	-0.6263	3.6365

Molecular structures of the considered Eu(III) and Cm(III) complexes of NTA, EDTA, and EGTA.

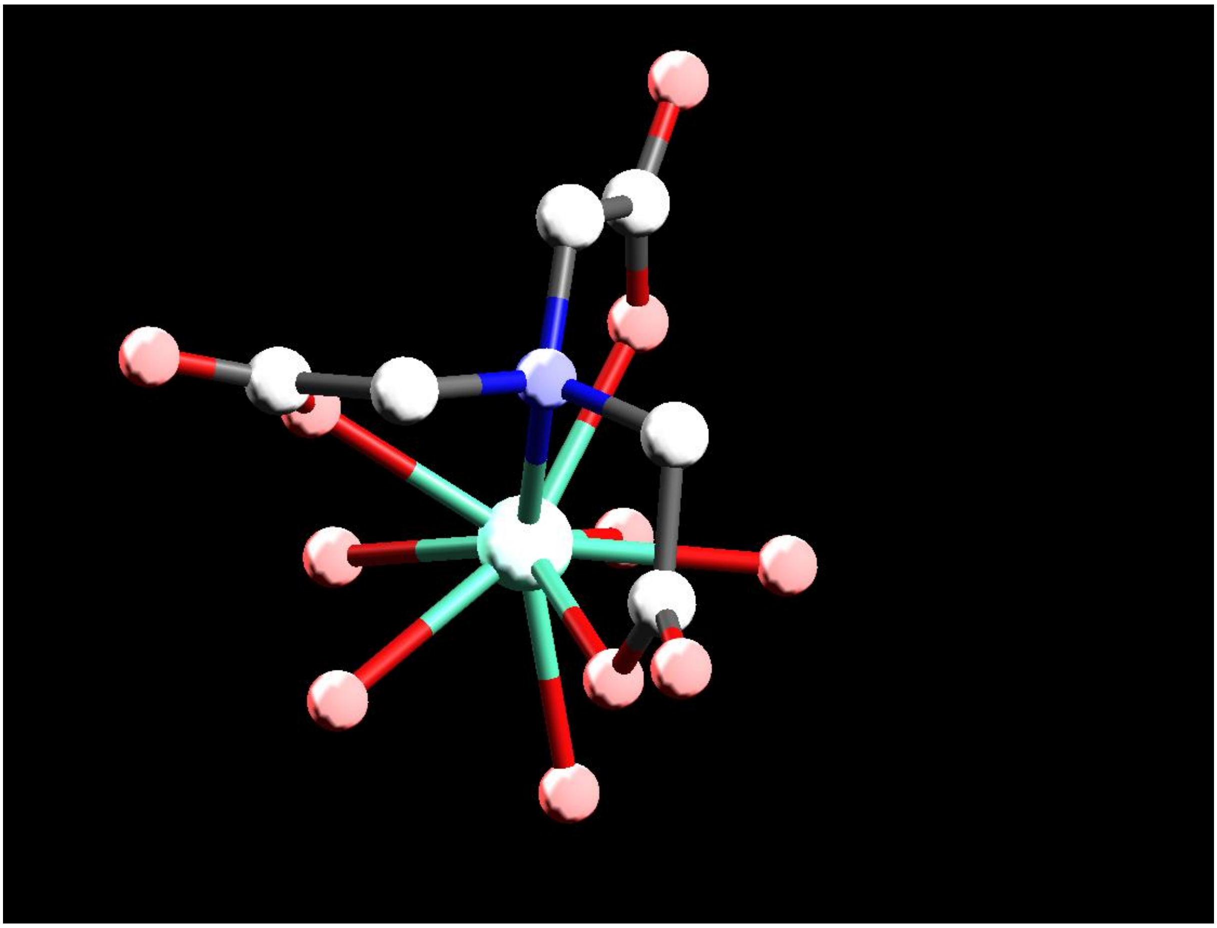

Figure S8. Representation of the molecular structure of the considered M–NTA 1:1 complex, M = Eu(III), Cm(III). Hydrogen atoms omitted for clarity; white, carbon; blue, nitrogen; red, oxygen; green, metal ion M.

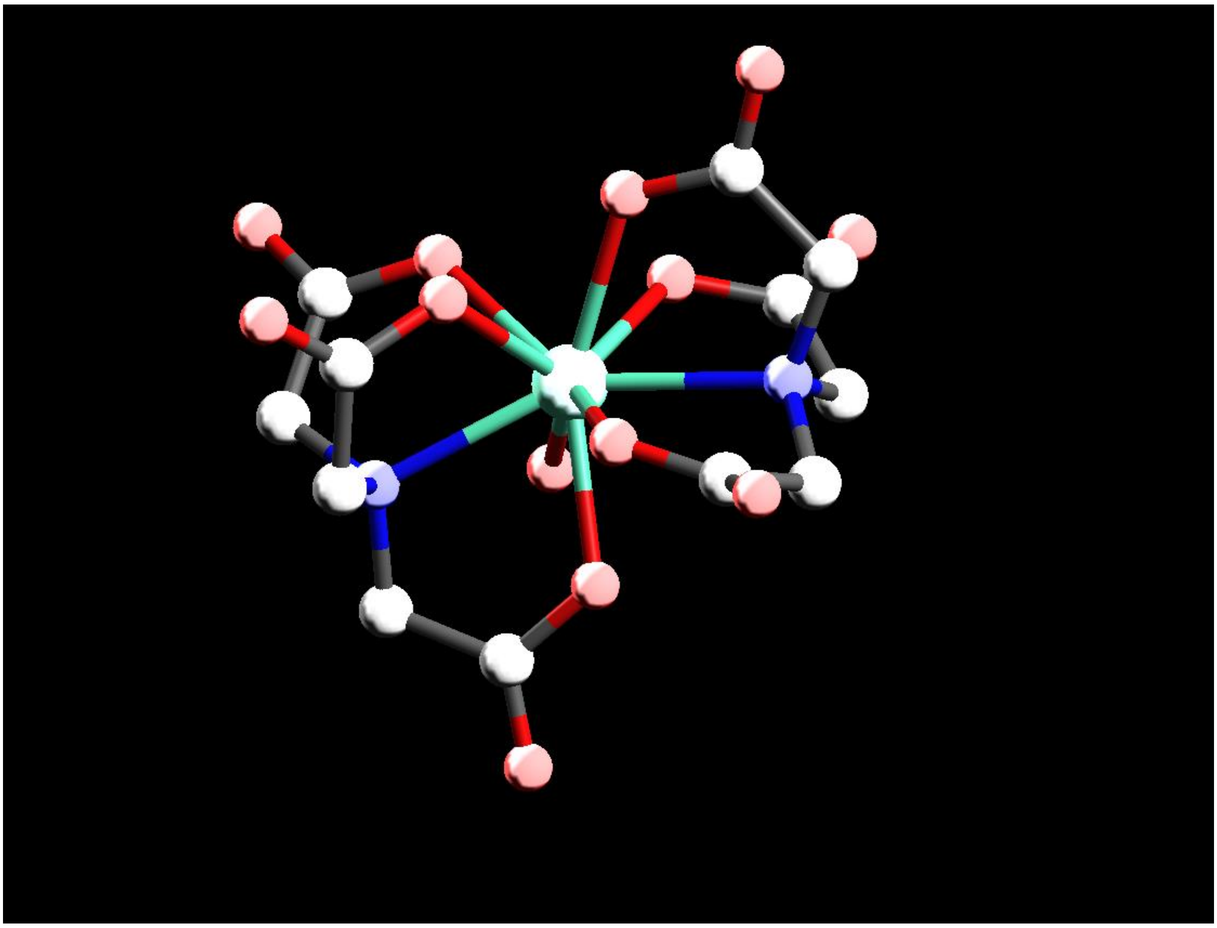

Figure S9. Representation of the molecular structure of the considered M–NTA 1:2 complex, M = Eu(III), Cm(III). Hydrogen atoms omitted for clarity; white, carbon; blue, nitrogen; red, oxygen; green, metal ion M.

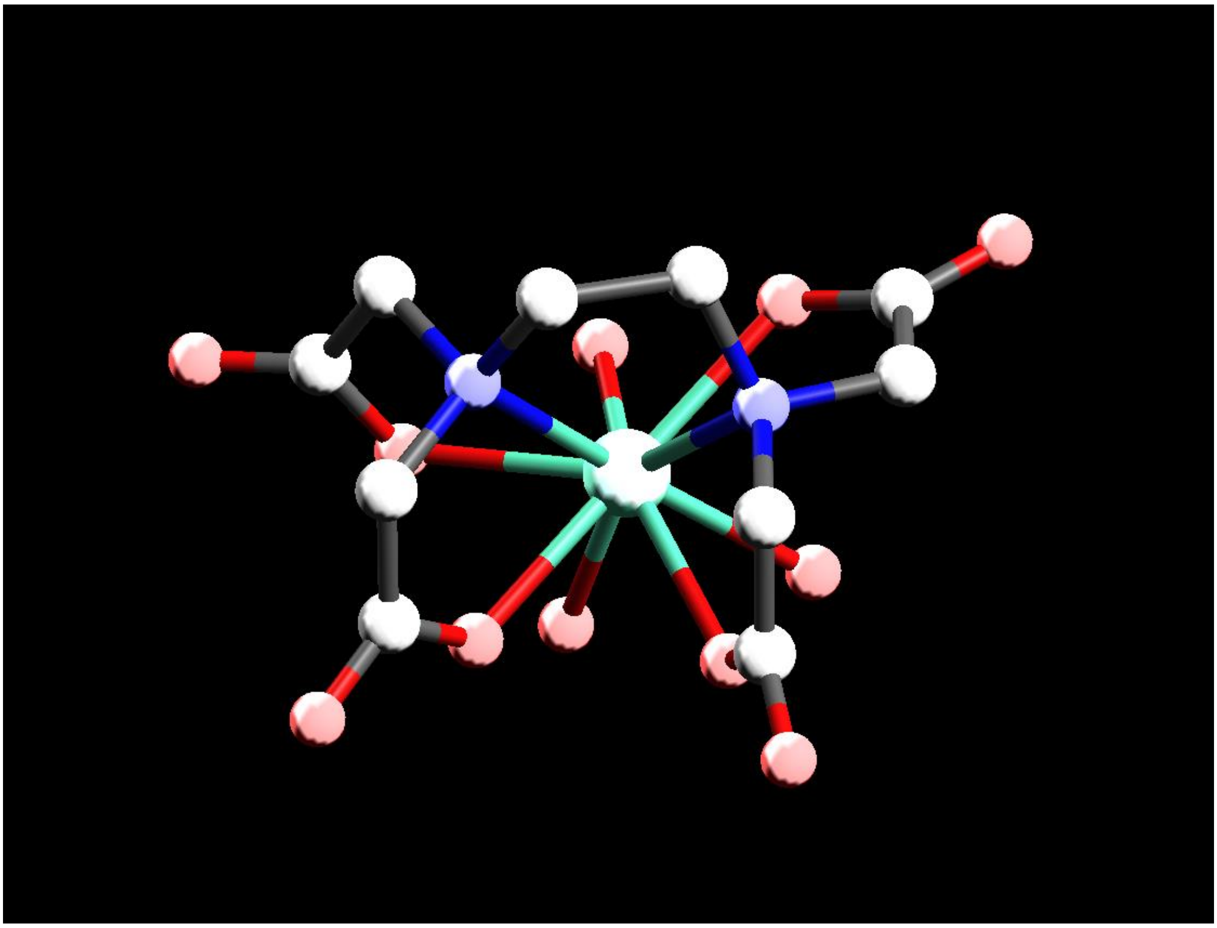

Figure S10. Representation of the molecular structure of the considered M–EDTA complex, M = Eu(III), Cm(III). Hydrogen atoms omitted for clarity; white, carbon; blue, nitrogen; red, oxygen; green, metal ion M.

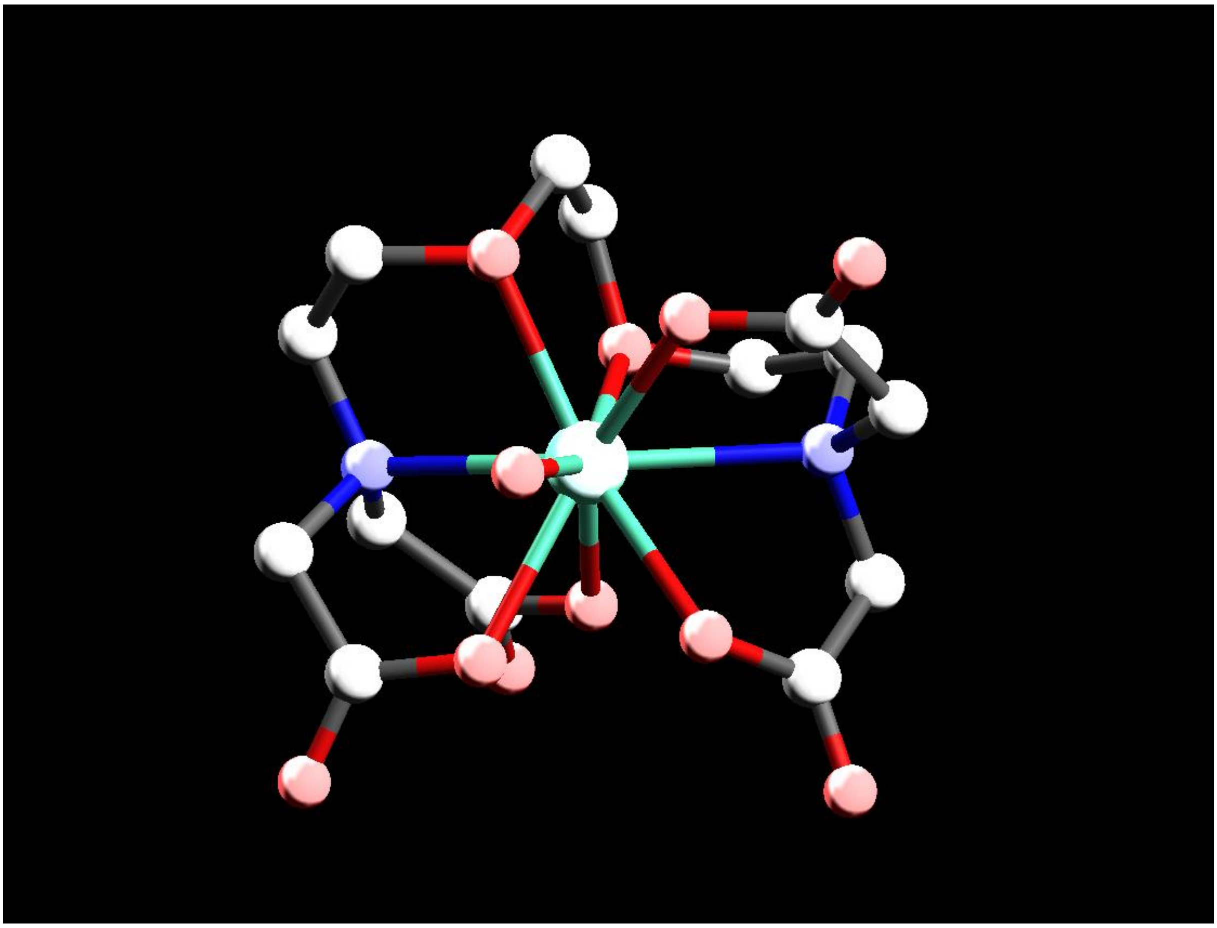

Figure S11. Representation of the molecular structure of the considered M–EGTA complex, M = Eu(III), Cm(III). Hydrogen atoms omitted for clarity; white, carbon; blue, nitrogen; red, oxygen; green, metal ion M.

Eu(III) complexation: time-resolved laser-induced luminescence spectra

NTA:

Eu(III)–NTA 2:2 complex-associated TRLFS characteristics

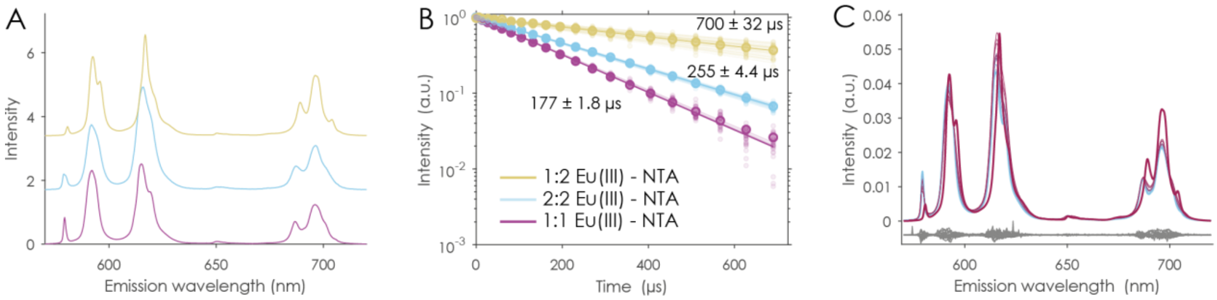

Figure S12. PARAFAC results for TRLFS spectra of Eu(III)–NTA 1:1, 1:2, and 2:2 complexes. Emission spectra (**A**), luminescence decays (**B**), and $^{7}F_{1}$ normalized emission spectra at t = 0 μs and the corresponding residuals (raw data − model) (**C**).

The three Eu–NTA complexes reveal distinct luminescence spectroscopic features. The individual emission spectra exhibit subtle but significant differences in shape and intensity for corresponding transitions. Especially the luminescence lifetime is a very good parameter for discrimination, and mirrors the decreasing number of coordinating water molecules in the 1:1, 2:2, and 1:2 complexes, respectively.

Eu(III)–NTA pH-series

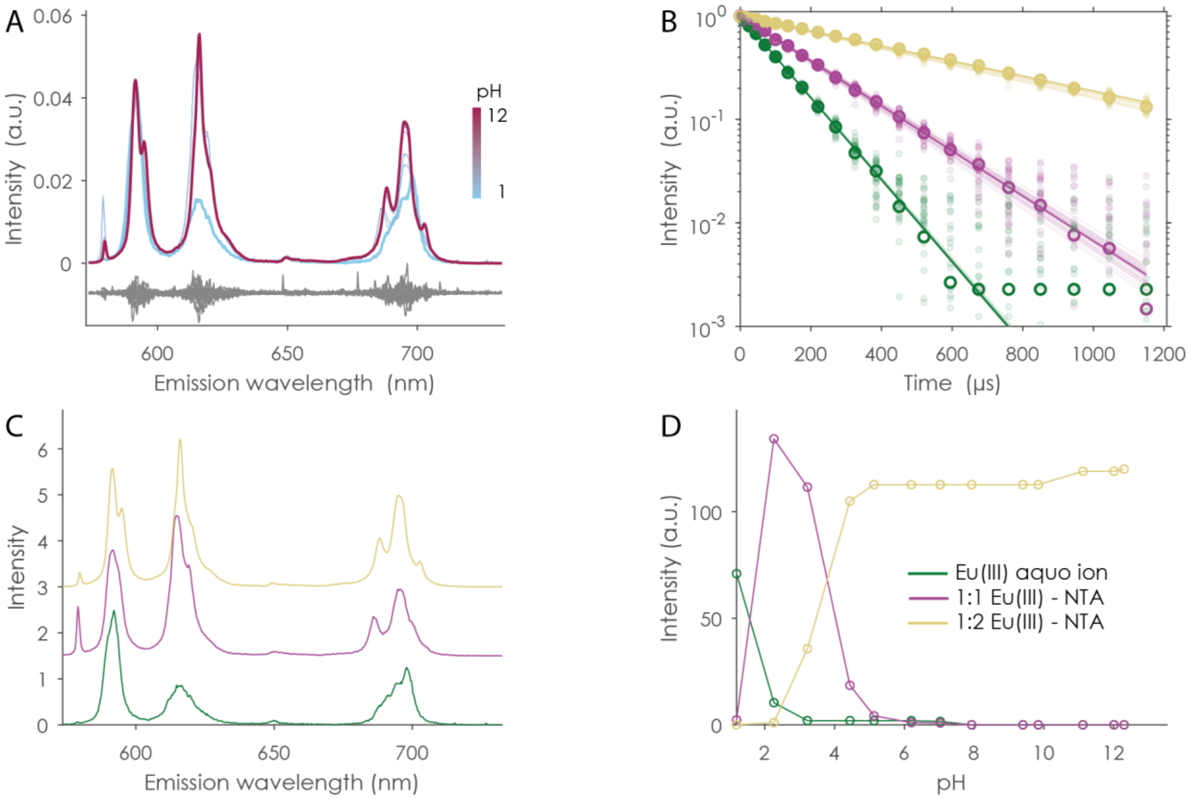

Figure S13. PARAFAC results for TRLFS series of the complexation of NTA with Eu(III) at varying pH. [Eu(III)] = 10 µM, [NTA] = 1 mM, [NaCl] = 0.1 M with pH varying from 1 to 12. $^7F_1$ normalized emission spectra at $t = 0$ µs and the corresponding residuals (raw data - model) (**A**), luminescence decays (**B**), emission spectra (**C**) and quantum yield-corrected PARAFAC distributions (symbols) and corresponding speciation (lines) (**D**).

Eu(III)–NTA concentration-series

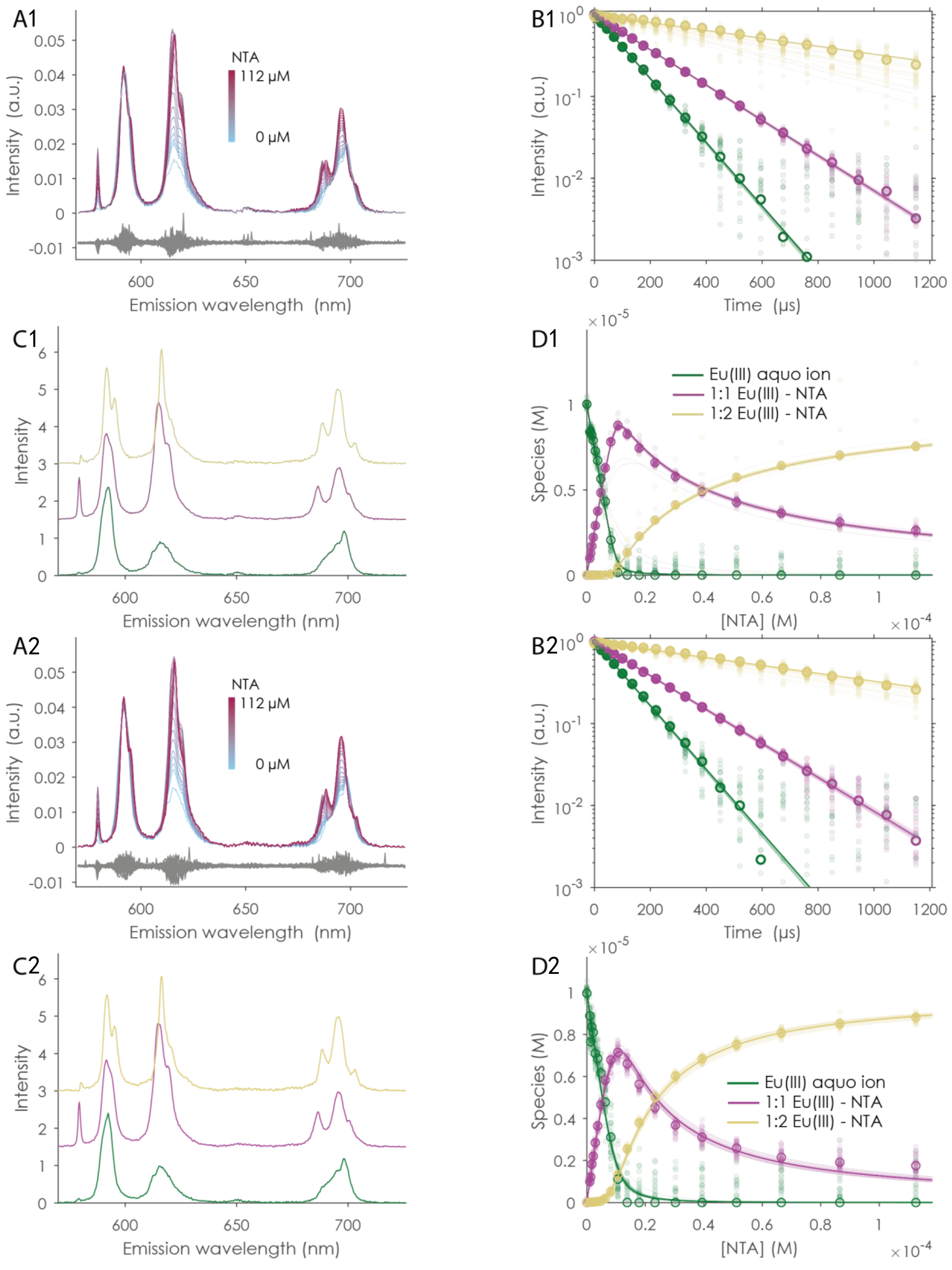

continued on next page

...continued

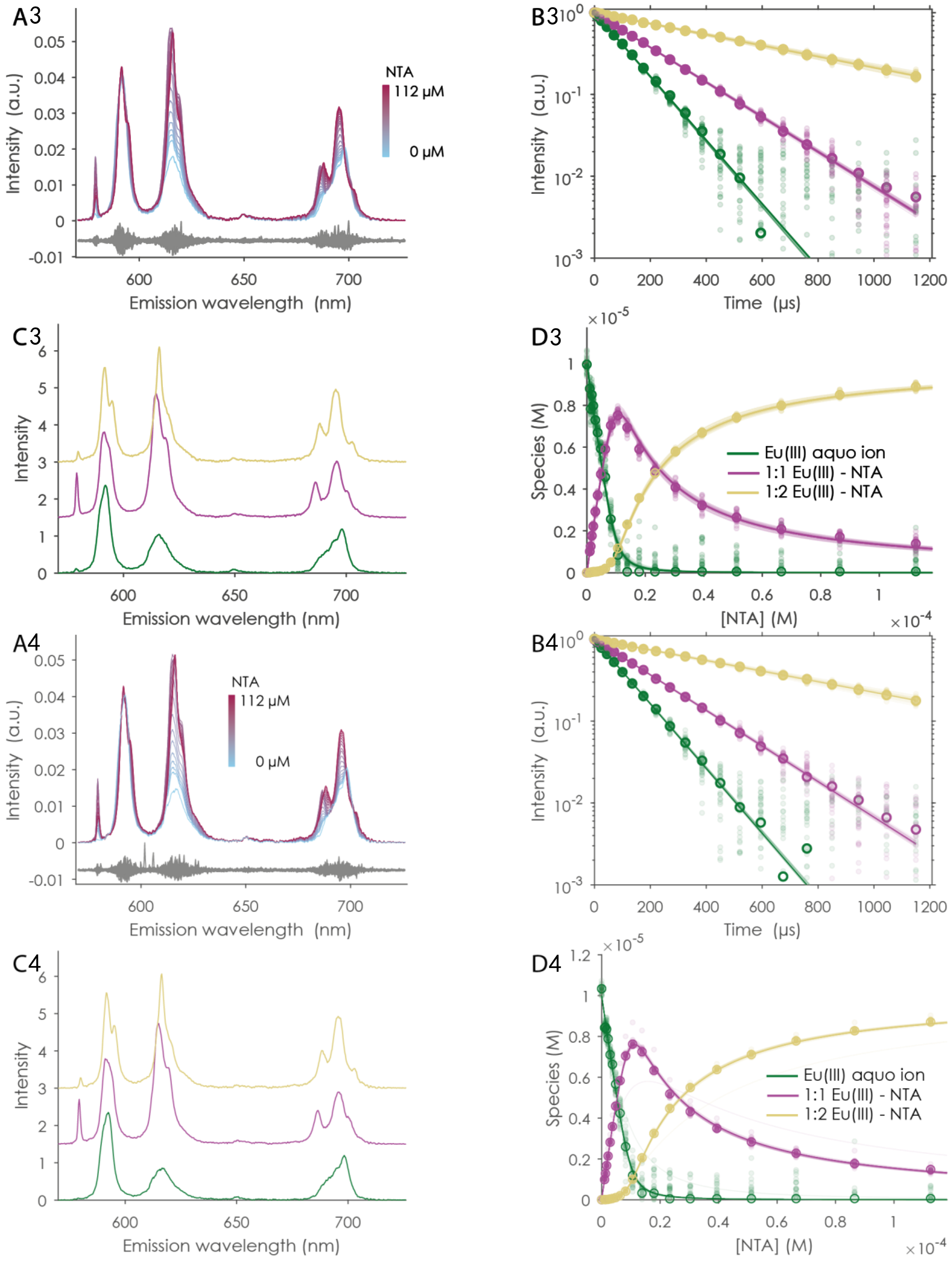

Figure S14. PARAFAC results of four independent TRLFS series of the complexation of NTA with Eu(III) at varying NTA concentration. [Eu(III)] = 10 μM, [NTA] = 0–112.5 μM, [NaCl] = 0.1 M, pH = 6.0. $^7F_1$ normalized emission spectra at t = 0 μs and the corresponding residuals (raw data − model) (**A1-A4**), luminescence decays (**B1-B4**), emission spectra (**C1-C4**), and quantum yield-corrected PARAFAC distributions (symbols) and corresponding speciation (lines) (**D1-D4**).

Cm(III)–NTA pH-series

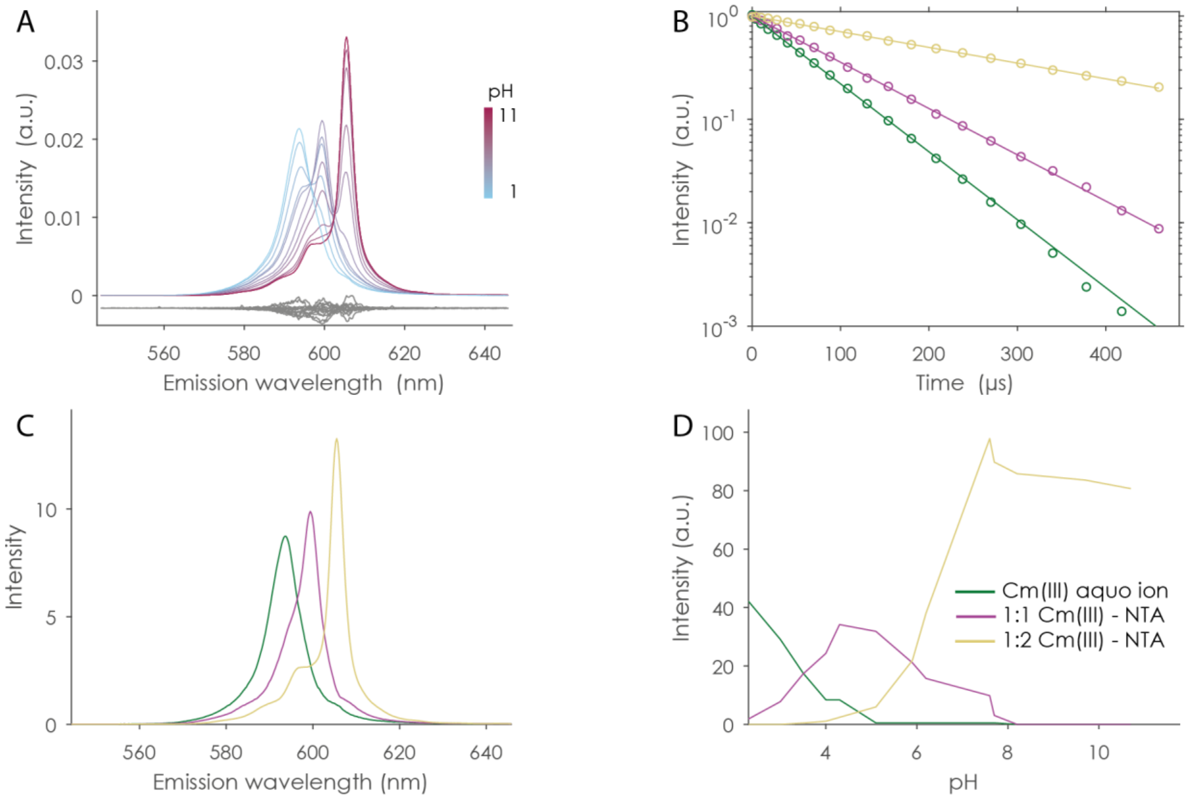

Figure S15. PARAFAC results for TRLFS series of the complexation of NTA with Cm(III) at varying pH. [Cm(III)] = 0.3 μM, [NTA] = 30 μM, [NaCl] = 0.1 M with pH varying from 1 to 11. Normalized emission spectra at $t = 0$ μs and the corresponding residuals (raw data − model) (**A**), luminescence decays (**B**), emission spectra (**C**), and quantum yield-corrected PARAFAC speciation (**D**).

EDTA:

Eu(III)–EDTA pH-series

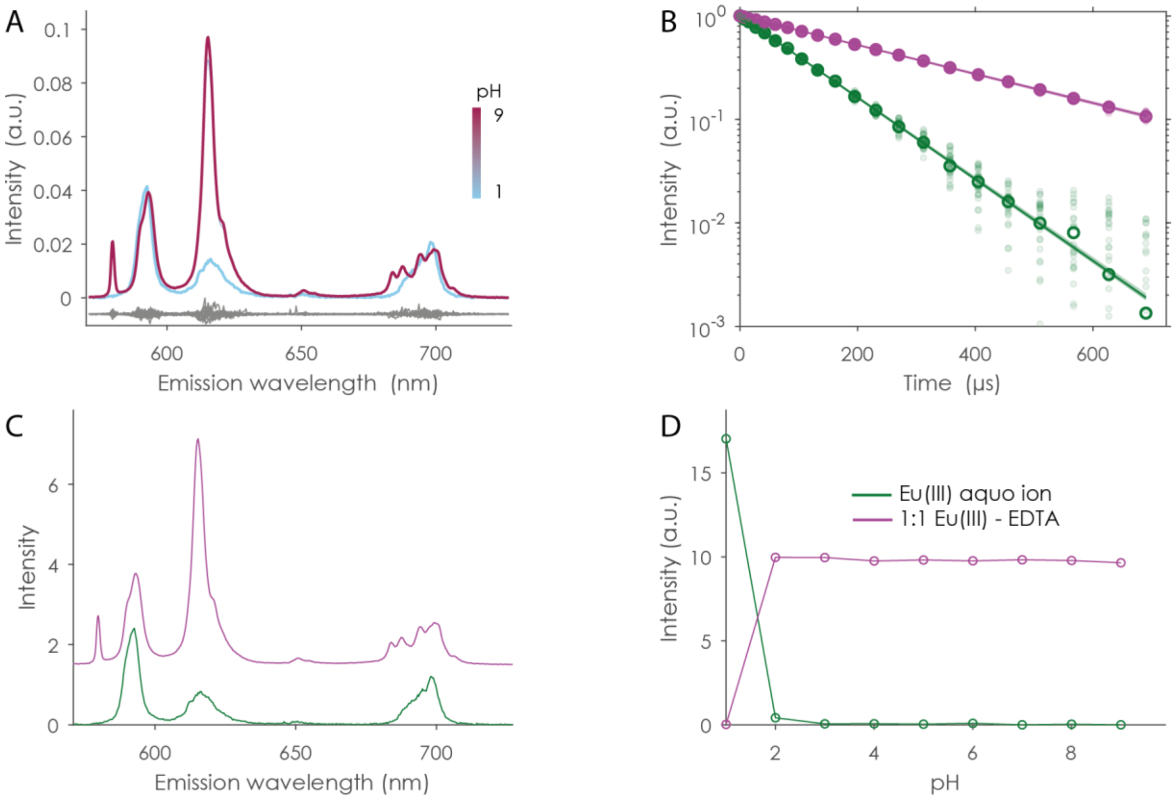

Figure S16. PARAFAC results for TRLFS series of the complexation of EDTA with Eu(III) at varying pH. [Eu(III)] = 10 μM, [EDTA] = 100 μM, [NaCl] = 0.1 M with pH varying from 1 to 9. $^7F_1$ normalized emission spectra at $t = 0$ μs and the corresponding residuals (raw data − model) (**A**), luminescence decays (**B**), emission spectra (**C**), and quantum yield-corrected PARAFAC distributions (symbols) and corresponding speciation (lines) (**D**).

Eu(III)–EDTA concentration-series

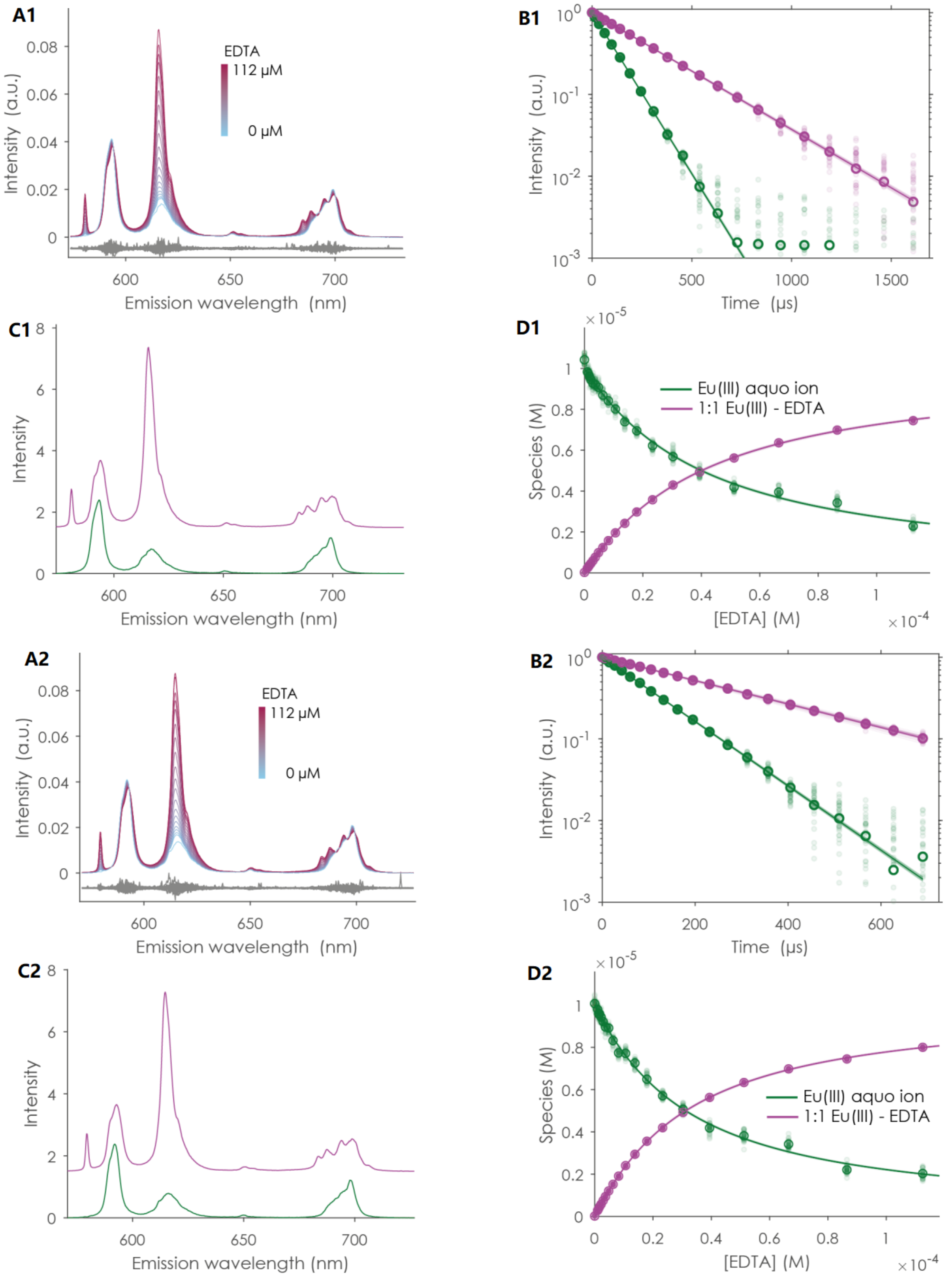

continued on next page

...continued

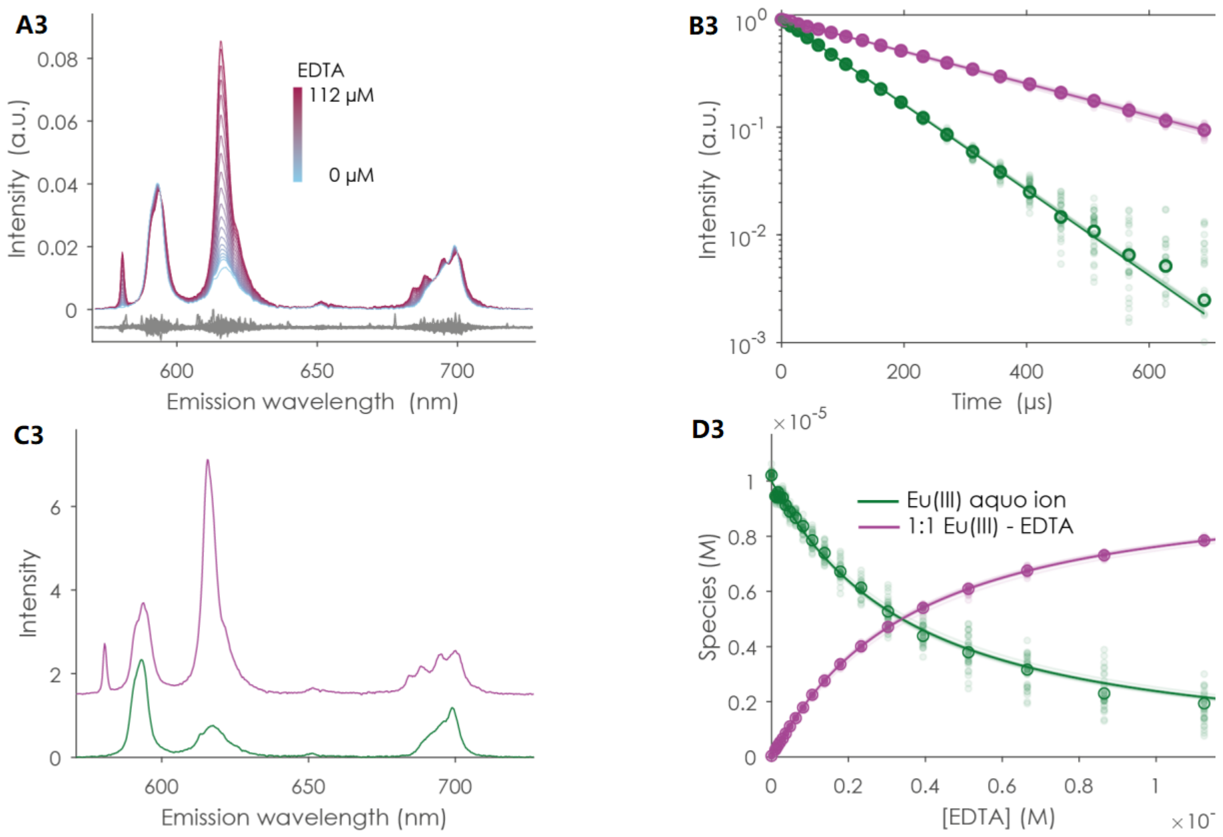

Figure S17. PARAFAC results of three independent TRLFS series of the complexation of EDTA with Eu(III) at varying EDTA concentration. [Eu(III)] = 10 µM, [EDTA] = 0–112.5 µM, [NaCl] = 0.1 M, pH = 2.4. $^7F_1$ normalized emission spectra at t = 0 µs and the corresponding residuals (raw data − model) (**A1-A3**), luminescence decays (**B1-B3**), emission spectra (**C1-C3**), and quantum yield-corrected PARAFAC distributions (symbols) and corresponding speciation (lines) (**D1-D3**).

EGTA:

Eu(III)–EGTA pH-series

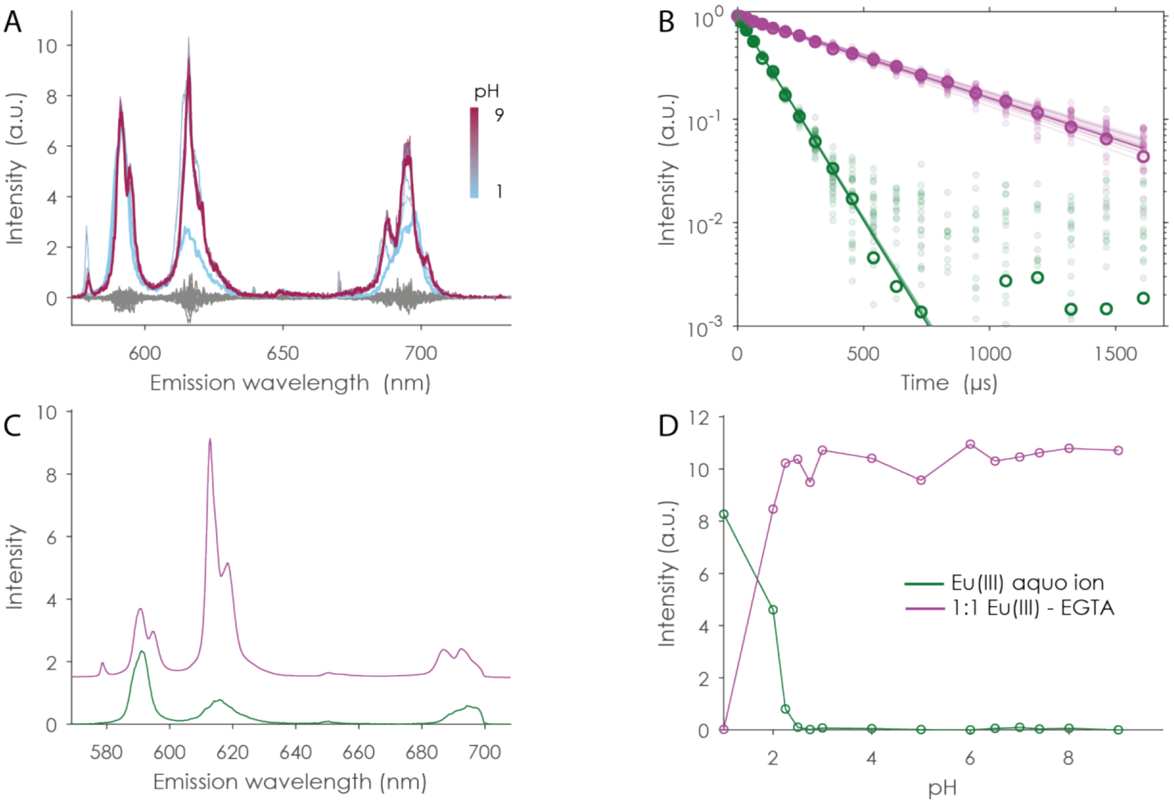

Figure S18. PARAFAC results for TRLFS series of the complexation of EGTA with Eu(III) at varying pH. [Eu(III)] = 10 μM, [EGTA] = 100 μM, [NaCl] = 0.1 M with pH varying from 1 to 9. $^7F_1$ normalized emission spectra at t = 0 μs and the corresponding residuals (raw data − model) (**A**), luminescence decays (**B**), emission spectra (**C**), and quantum yield-corrected PARAFAC distributions (symbols) and corresponding speciation (lines) (**D**).

Eu(III)–EGTA concentration-series

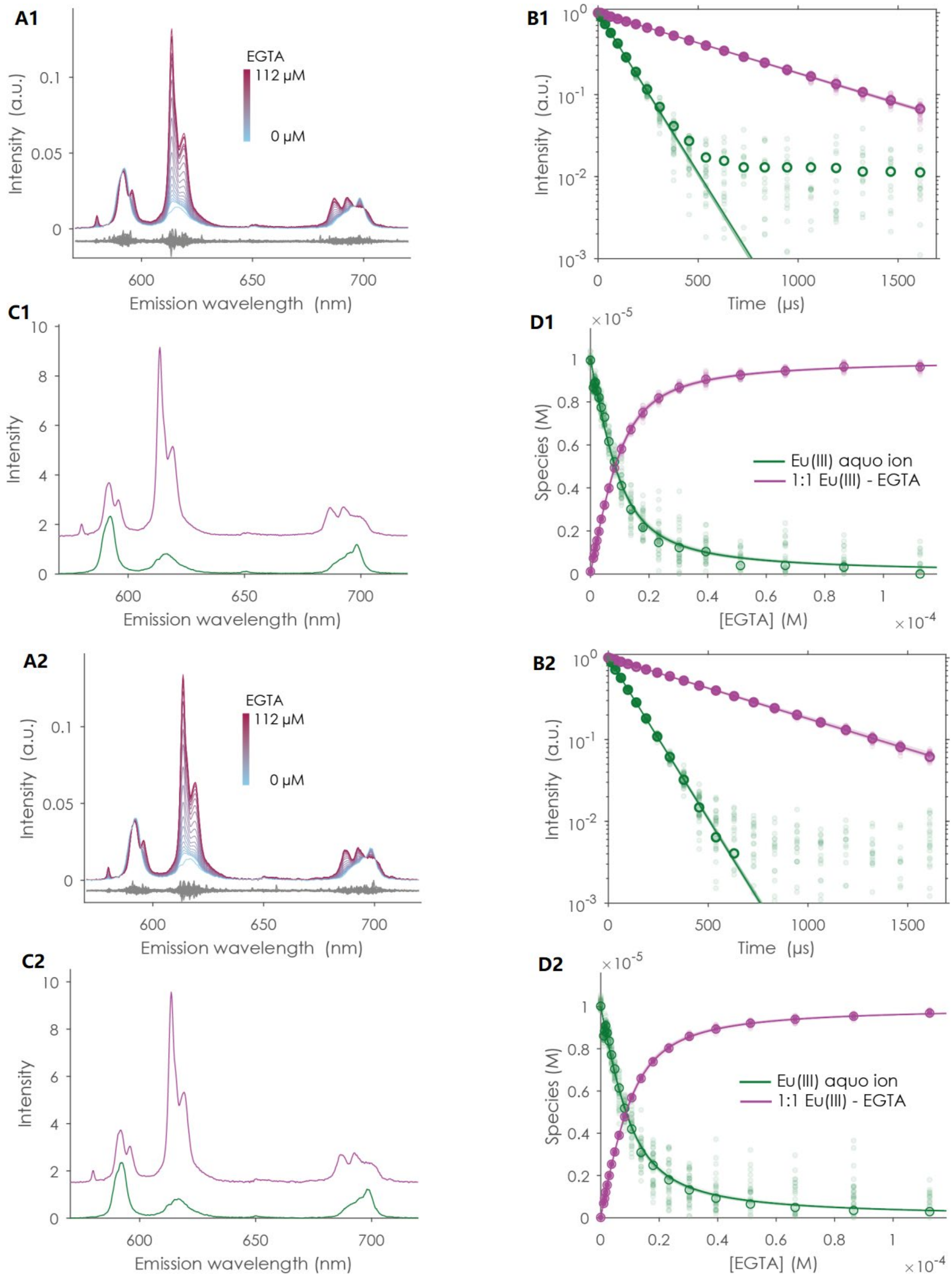

continued on next page

… continued

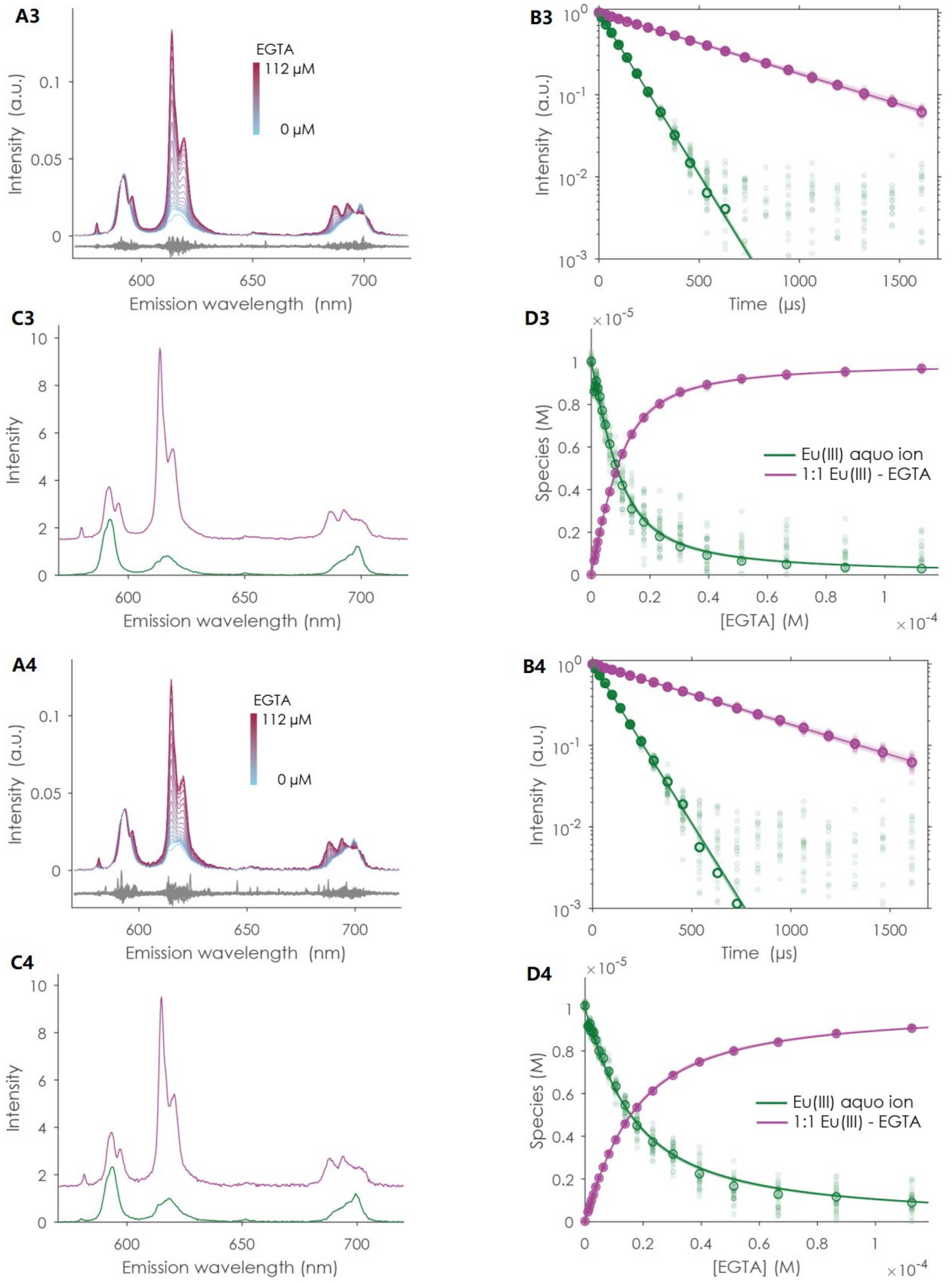

Figure S19. PARAFAC results of four independent TRLFS series of the complexation of EGTA with Eu(III) at varying EGTA concentration. [Eu(III)] = 10 µM, [EGTA] = 0–112.5 µM, [NaCl] = 0.1 M, pH = 3.0. $^7F_1$ normalized emission spectra at t = 0 µs and the corresponding residuals (raw data − model) (**A1-A4**), luminescence decays (**B1-B4**), emission spectra (**C1-C4**), and quantum yield-corrected PARAFAC distributions (symbols) and corresponding speciation (lines) (**D1-D4**).

Cm(III)–EGTA pH-series

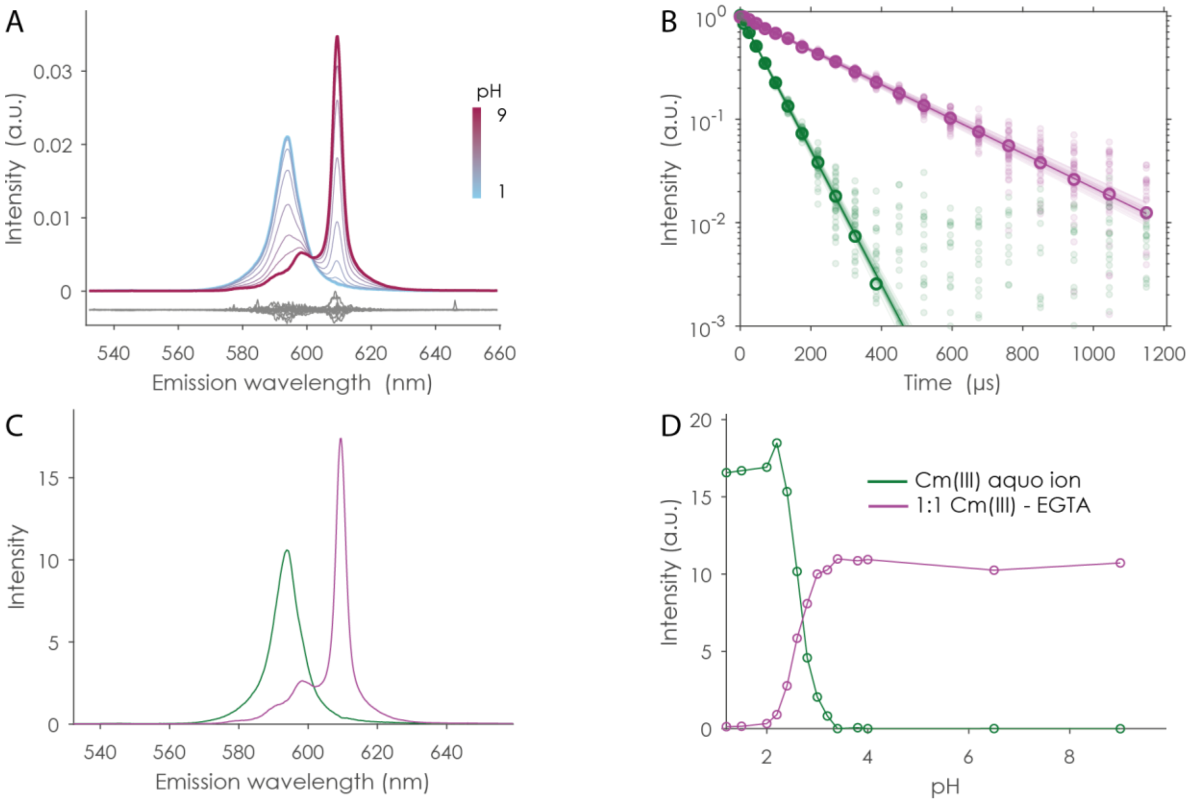

Figure S20. PARAFAC results for TRLFS series of the complexation of EGTA with Cm(III) at varying pH. [EGTA(III)] = 0.3 µM, [NTA] = 30 µM, [NaCl] = 0.1 M with pH varying from 1 to 9. Normalized emission spectra at $t = 0$ µs and the corresponding residuals (raw data − model) (**A**), luminescence decays (**B**), emission spectra (**C**), and quantum yield-corrected PARAFAC speciation (**D**).

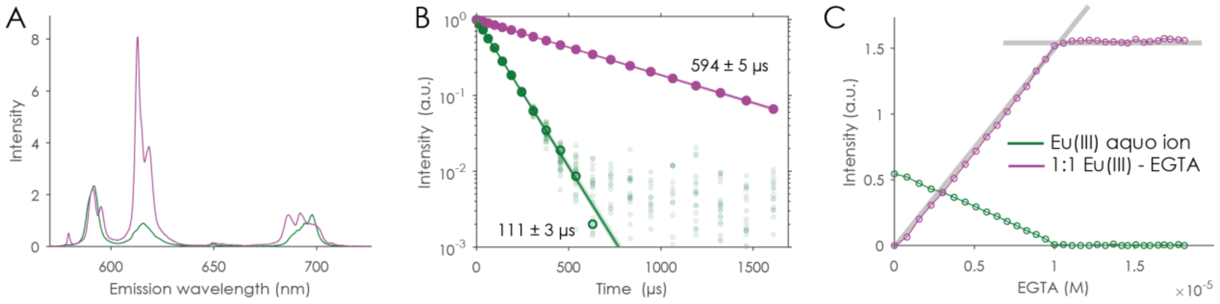

Figure S21. PARAFAC results for TRLFS series of the complexation of EGTA with Eu(III) at varying EGTA. [Eu(III)] = 10 µM, [EGTA] = 0–17 µM, [NaCl] = 0.1 M, pH = 6.0. Emission spectra (**A**), luminescence decays (**B**), and quantum yield-corrected PARAFAC distributions (symbols) and corresponding speciation (lines) (**C**).

Eu(III) complexation: isothermal titration calorimetry

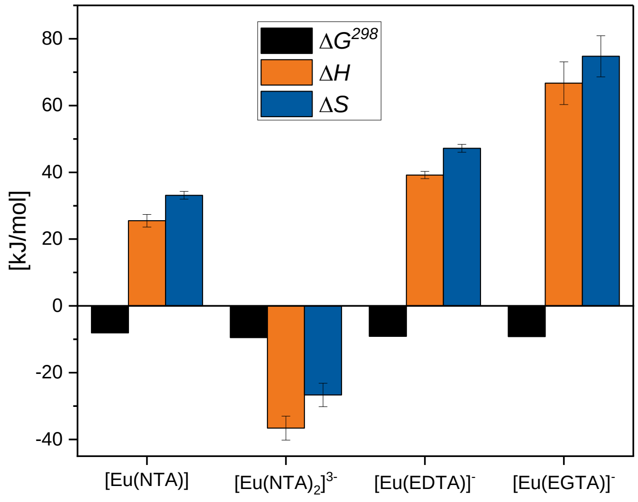

Figure S22. ITC results for all observed complexes with contributions from Δ*H*, Δ*S*, and ΔG^{298}.

Table S1. Logarithmic complex formation constant individual data of TRLFS measurements of Eu(III) complexes with NTA, EDTA, and EGTA, obtained from triplicate experiments.

Species	Run		
	1	2	3
$[Eu(NTA)]^0_{aq}$	11.7	11.5	11.4
$[Eu(NTA)_2]^{3-}$	20.6	20.4	20.4
$[Eu(EDTA)]^-$	17.0	16.9	17.0
$[Eu(EGTA)]^-$	18.0	18.0	17.7

Table S2. Thermodynamic parameters individual data of ITC measurements of Eu(III) complexes with NTA, EDTA, and EGTA.

Species	Run	$[Eu(NTA)]^0_{aq}$	$[Eu(NTA)_2]^{3-}$	$[Eu(EDTA)]^-$	$[Eu(EGTA)]^-$
log *β*	1	11.4	20.0	17.0	18.1
	2	11.5	20.3	17.0	17.7
	3	11.4	20.2	17.0	18.1
	4			17.0	
Δ*H* (kJ/mol)	1	27.4	−40.7	39.8	61.2
	2	24.3	−38.0	37.0	76.2
	3	25.0	−31.2	39.8	62.6
	4			40.3	
Δ*S* (J/mol·K)	1	119	−105	164	236
	2	109	−96	155	286
	3	111	−73	164	241
	4			166	
ΔG^{298} (kJ/mol)	1	−8.1	−9.5	−9.1	−9.3
	2	−8.1	−9.5	−9.1	−9.2
	3	−8.1	−9.5	−9.1	−9.3
	4			−9.1	

Reference

1 Aime, S.; Barge, A.; Borel, A.; Botta, M.; Chemerisov, S.; Merbach, A.E.; Müller, U.; Pubanz, D. A multinuclear NMR study on the structure and dynamics of lanthanide(III) complexes of the poly(amino carboxylate) EGTA4− in aqueous solution. *Inorg. Chem.* **1997**, *36*, 5104–5112
